# Methylphenidate modulates interactions of anxiety with cognition

**DOI:** 10.1038/s41398-021-01621-2

**Published:** 2021-10-21

**Authors:** C. Gaillard, T. R. Lago, A. X. Gorka, N. L. Balderston, B. A. Fuchs, R. C. Reynolds, C. Grillon, M. Ernst

**Affiliations:** 1 Section on Neurobiology of Fear and Anxiety, National Institute of Mental Health, Bethesda, MD USA; 2 Department of Psychiatry, Boston University School of Medicine, Boston, MA USA; 3 Center for Neuromodulation in Depression and Stress, Department of Psychiatry, University of Pennsylvania, Philadelphia, PA USA; 4 Department of Nutritional Sciences, The Pennsylvania State University, University Park, PA USA; 5 Scientific and Statistical Computing Core, National Institute of Mental Health, Bethesda, MD USA

**Keywords:** Psychiatric disorders, Human behaviour

## Abstract

While a large body of literature documents the impairing effect of anxiety on cognition, performing a demanding task was shown to be effective in reducing anxiety. Here we explored the mechanisms of this anxiolytic effect by examining how a pharmacological challenge designed to improve attentional processes influences the interplay between the neural networks engaged during anxiety and cognition. Using a double-blind between-subject design, we pharmacologically manipulated working memory (WM) using a single oral dose of 20 mg methylphenidate (MPH, cognitive enhancer) or placebo. Fifty healthy adults (25/drug group) performed two runs of a WM *N*-back task in a 3 T magnetic resonance imaging scanner. This task comprised a low (1-Back) and high (3-Back) WM load, which were performed in two contexts, safety or threat of shocks (induced-anxiety). Analyses revealed that (1) WM accuracy was overall improved by MPH and (2) MPH (vs. placebo) strengthened the engagement of regions within the fronto-parietal control network (FPCN) and reduced the default mode network (DMN) deactivation. These MPH effects predominated in the most difficult context, i.e., threat condition, first run (novelty of the task), and 3-Back task. The facilitation of neural activation can be interpreted as an expansion of cognitive resources, which could foster both the representation and integration of anxiety-provoking stimuli as well as the top–down regulatory processes to protect against the detrimental effect of anxiety. This mechanism might establish an optimal balance between FPCN (cognitive processing) and DMN (emotion regulation) recruitment.

## Introduction

While a large body of literature documents the impairing effect of anxiety on cognition [for reviews, see [1–4], performing a demanding task was shown to be effective in reducing anxiety [5–10]. This neuroimaging study examines potential neural mechanisms underlying this effect that could be exploited in the treatment of anxiety. To this aim, a pharmacological manipulation of cognitive function using a cognitive enhancer (methylphenidate (MPH)) was designed to investigate the interaction of induced-anxiety with performance on a working memory (WM) task.

Heuristic models of anxiety provide strong anchors for the formulation of hypotheses in which cognitive mechanisms are prominent [8, 9, 11–13]. For example, the famous revised model of WM elaborated by Baddeley et al. [14, 15] provides a mechanistic framework based on cognitive resources to account for anxiety. Most importantly, this model communicates the principle of limited cognitive capacity, which, when insufficient, results in behavioral perturbations [12, 13]. Indeed, finite cognitive capacity leads to competition between processes, favoring the processing of the most biologically salient behavior [4, 13]. In contrast to this well-recognized theory, a recent behavioral study conducted in our laboratory suggested that, in fact, cognitive capacity may not be finite [16]. This behavioral study was designed to test interactions of anxiety and cognition, as each was manipulated separately. MPH, a cognitive enhancer (20 mg PO) was expected to facilitate cognitive performance (*N*-Back WM) through the prioritization of cognitive processes and at the expense of the processing of anxiety. Anxiety was induced by unpredictable threat of shock. Compared to placebo (PLA), MPH administration alleviated the impairing effect of induced-anxiety on WM accuracy at the highest cognitive load (i.e., 3-Back task). At the same time, MPH was associated with stronger physiological responses (startle reflex) to anxiety (threat of shock). These findings suggested that cognitive resources could be used to both foster the engagement of cognitive capacity and to process anxiety-provoking stimuli and associated defensive responses. In the present study, we wished to test this hypothesis against the classical limited resources model, at the neural level.

**Fig. 1:**
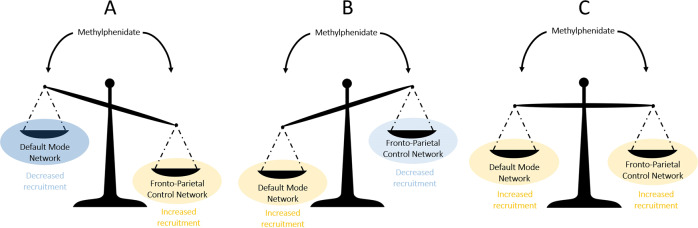
Schematics of the three predictions underpinning MPH-mediated cognitive enhancement. **A** MPH-driven increase in the dissociation between the engagement of the FPCN and DMN as reflected by (i) stronger activation of fronto-parietal control network (FPCN) regions (task-positive network), which favors cognitive performance and (ii) steeper deactivation of default mode network (DMN) regions (task-negative network), which prevents interference from irrelevant stimuli such as anxiety-related signals. **B** MPH-driven redistribution of cognitive resources as indicated by lower resource allocation to the FPCN regions (weaker activation), which evidences greater efficiency of the executive control system. Consequently, more resources are available to process and inhibit anxiety, which might be further reflected by increased activation of the DMN regions. **C** MPH-driven expansion of overall resources as reflected by stronger activation of both the FPCN regions (i.e., better cognitive performance and inhibition of interference from non-relevant stimuli) and the DMN regions (i.e., processing of anxiety without interfering with cognitive performance).

Two neural networks play an essential role regarding the impact of cognition on anxiety, the fronto-parietal control network (FPCN) and the default mode network (DMN). Cognitive function, such as WM, activates the FPCN (task-positive network) and deactivates the DMN (task-negative network) [for a review, see [17]]. Anxiety alters both the FCPN and the DMN, in ways that depend on the context [18, 19], for reviews, see [20, 21]. The most common effects of anxiety on neural networks, evidenced by resting-state neuroimaging studies, are of two types: (1) an impaired functional connectivity of both the FPCN (responsible for processes of attention, control, supervision) and DMN (responsible for self-referential processes), and (2) an increased functional connectivity of the salience network (responsible for coding biological significance of stimuli) and the bottom–up attention network (i.e., ventral attention network, responsible for stimulus-driven attention processes) [for a review, see [21]]. Additionally, anxiety is associated with a hypoconnectivity between the affective network and both the FPCN and DMN, as well as a decoupling between the FPCN and the DMN [for a meta-analytic review, see [20]]. Here we propose a theoretical framework based on a relative functional balance between FPCN and DMN. This framework provides three possible alternate mechanisms underlying the relation between cognitive enhancement and anxiety reduction (see Fig. 1): (A) MPH increases the dissociation between the engagement of the FPCN and DMN, as reflected in the combination of (i) stronger activation of FPCN regions (task-positive network), which favors cognitive performance, and (ii) steeper deactivation of DMN regions (task-negative network), which prevents interference from irrelevant stimuli such as self-referential thoughts (worry) [for a review, see [20]]. (B) MPH permits a redistribution of cognitive resources as indicated by lower resource allocation to the FPCN (weaker activation), which signals MPH-related increased efficiency of the executive control system [22, 23], leaving more resources to process and inhibit anxiety (i.e., increased DMN activation). (C) MPH expands overall neural resources as reflected by stronger activation of both the FPCN (i.e., better cognitive performance and inhibition of interference from non-relevant stimuli) and the DMN (i.e., processing of anxiety without interfering with cognitive performance and allowing regulatory control of anxiety) [16].

Finally, consistent with previous works [e.g., [24, 25], we expected that MPH-driven effect would be detected most reliably in the task condition that requires the most resources, i.e., at the highest WM load, during induced-anxiety (compared to safety), and when the task is novel to the participants (beginning vs. later in the WM performance).

## Materials and methods

### Participants

Seventy healthy volunteers were recruited from the Washington DC metropolitan area. Sample size was determined prior to recruitment based on our previous psychopharmacological study using MPH conducted in the clinic [16]. A comprehensive clinical (medical history and exam) and psychiatric [SCID I/NP; [26]] screen confirmed criteria for study participation. Inclusion criteria were: (a) age between 18 and 50 years; (b) no past or current Axis I psychiatric disorders, (c) no family history of psychiatric disorder in first-degree relatives, (d) no significant medical or neurological conditions that might interfere with participation in the study, (e) no use of psychotropic medications, tobacco, or illicit drugs (urine screen), and (f) no contraindications to magnetic resonance imaging (MRI). In addition, exclusion was based on the following criteria: (a) prior chronic use of MPH such as Ritalin, (b) intelligence quotient (IQ) <80 as assessed using the vocabulary and matrix reasoning subscales of the Wechsler Abbreviated Scale of Intelligence [WASI; [27]], (c) pregnancy or a positive pregnancy test. Twenty participants were excluded from analyses due to missing functional MRI (fMRI) sequences (*n* = 4), incomplete behavioral data (*n* = 6), and excessive head motion (*n* = 10). The final sample consisted of 50 healthy, right-handed adults (26 females; age mean = 28.2 years, SD = 6.9 years). Demographic information of the two groups is presented in Table S1. Prior to participation, all subjects signed consent form approved by the National Institute of Mental Health Combined NeuroScience Institutional Review Board and were compensated for their time. Study procedure is detailed in Supplemental Material.

### Drug administration

A randomized double-blind parallel-group design was used in this PLA-controlled study. Participants were randomly assigned to receive either a single oral dose of PLA or immediate-release MPH 20 mg (Ritalin, Novartis, Basel, Switzerland), according to a randomization schedule established by the National Institutes of Health pharmacy. PLA and MPH oral doses were presented in identical-appearing capsules. The MPH dose was selected based on the lowest dose reported to be effective on cognitive function [22, 28, 29]. Plasma levels of MPH have been reported to peak 1–2 h after drug administration, with a plasma half-life ranging from 1 to 4 hr [30, 31]. Therefore, drug was administered 90 min prior to beginning the experimental WM task in the scanner [22, 28, 32]. Potential side effects and adverse reactions were monitored using a clinician-administered 34-item Adverse Events Checklist.

### *N*-Back WM task

**Fig. 2:**
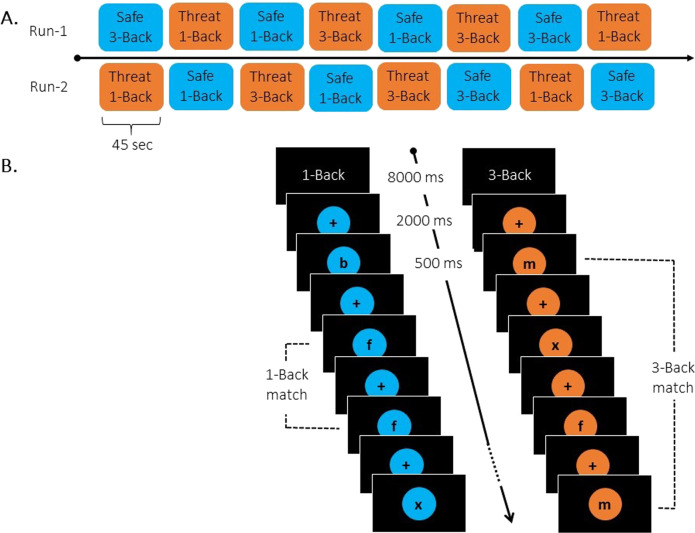
Schematic of the *N*-Back working memory (WM) task. **A** Illustration of the two runs, one starting with a Threat block, and the other with a Safe block. The order of runs was counterbalanced across participants. In addition, participants performed the task in two different contexts, safety (Safe blocks) or threat of electrical shock (Threat blocks). Safe and Threat blocks alternated within each run and were paired with difficulty (i.e., 1-Back or 3-Back task). Each run comprised 8 blocks (45 s each) in total, with 2 blocks per task condition (i.e., 1-Back/Safe, 1-Back/Threat, 3-Back/Safe, 3-Back/Threat). **B** Illustration of a Safe block (letters surrounded by blue color) of a 1-Back task and a Threat block (letters surrounded by orange color) of a 3-Back task. At the beginning of each block, an instruction screen (8000 ms) notified participants of task load, i.e., 1-Back or 3-Back task. Within each block, 18 letters were sequentially presented for 500 ms at 2000 ms interval. Prior to the experiment, participants were instructed that, during the Safe blocks (letters surrounded by blue color), they would never receive an electrical shock, whereas unpredictable electrical shocks could be delivered at any time during the Threat blocks (letters surrounded by orange color). In total, six shocks were delivered throughout the task, three in each run.

**Fig. 3:**
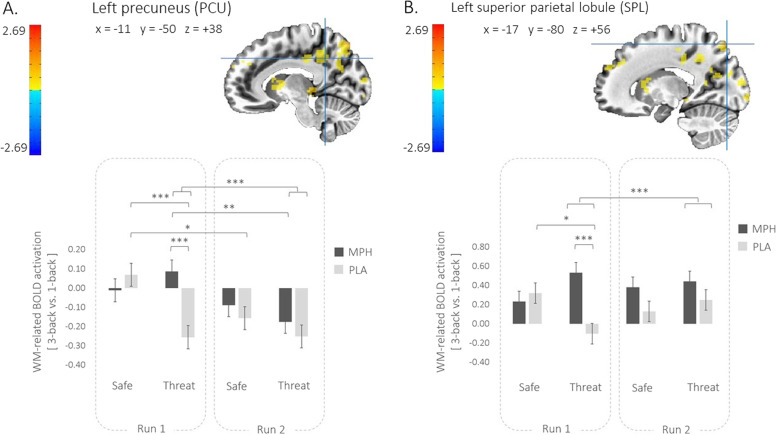
Interaction effect of *Drug*, *Run*, and *Condition* on WM-related BOLD activation in left precuneus (PCU) and left superior parietal lobule (*SPL*). Upper panel: *Drug* by *Run* by *Condition* interaction on WM-related BOLD (3-Back vs. 1-Back contrast) recruitment of the **A** left PCU and **B** left SPL. Lower panel: Histograms of the parameter estimates extracted from the **A** left PCU and **B** left SPL. Linear mixed-effects models were conducted on the parameter estimates with *Drug*, *Run*, and *Condition* as fixed factors, and *Subjects* as random factor. *Sex* and *Age* were included as covariates. Error bars represent mean ± standard error of the mean. Color bar: warm colors represent WM-related BOLD activation in MPH compared to PLA, while cool colors represent WM-related BOLD activation decrease in MPH compared to PLA. Crosshairs depict the location of the voxel with the peak activation for the given cluster. Wholebrain activations are corrected for multiple comparisons using a cluster-based approach with a voxel-wise bi-sided *p*-value threshold of *p* < 0.001 and a minimum cluster size of *k* = 16 (432 μL), which corresponds to a cluster-level alpha of *p* < 0.05 using NN2 clustering, where clustered voxels can share faces and edges. For visualization purpose, whole-brain activationsare thresholded here at *p* = 0.01. *p < 0.05, **p < 0.01, ***p < 0.001.

The *N*-Back WM task consisted of single letters displayed sequentially (see Fig. 2). Participants were asked to press a button to indicate whether the letter currently presented on the screen was identical to (matched) or different from (did not match) the letter presented *N* letters before. Based on previous studies using the *N*-Back WM task [e.g., [16, 33, 34]], 33% of the letters were identical to the letter presented *N* letters before. Two levels of difficulty were tested: 1-Back and 3-Back. An instruction screen (8000 ms) appeared at the beginning of each block to signal the upcoming level of the WM load. The task was organized into two runs with Run-2 starting 10 min after Run-1. Each run consisted of 8 blocks of 18 letters each (see Supplemental Material for additional details related to stimuli and apparatus). Letters were presented for 500 ms at 2000 ms interval. In each run, blocks alternated between periods of safety (Safe blocks) and threat of unpredictable electrical shocks (Threat blocks) (see Fig. 3A). Each Safe and Threat block was paired with a level of difficulty (1-Back, 3-Back), so that each run contained two blocks per conditions (i.e., 1-Back/Safe, 3-Back/Safe, 1-Back/Threat, 3-Back/Threat). The color surrounding the letter presented on the screen signaled a Safe block (blue) or a Threat block (orange). Participants were instructed that they would never receive an electrical shock during the Safe blocks (blue) but that they could receive unpredictable electrical shocks at any time during the Threat blocks (orange). In total, six shocks were delivered throughout the task, three per run (see Supplemental Material for additional details related to shock intensity calibration).

### Anxiety subjective ratings

Subjective anxiety was assessed from two perspectives, general anxiety state across the whole study visit and anxiety in the fMRI, specifically linked to task performance. The State-Trait Anxiety Inventory [STAI-s; [35]] monitored general state anxiety at three time points: (1) STAI-s 1, right after drug administration (T1), when the pharmacological action of the drug had not yet started to take effect, (2) STAI-s 2, 45 min post drug administration. At this time, MPH was expected to start having clinical effects (T2). Finally, (3) STAI-s 3, as soon as participants were removed from the scanner (T3). Information related to anxiety assessment during fMRI are presented in Supplemental Material.

### fMRI data acquisition and analysis

#### fMRI data acquisition

Two runs of 225 multi-echo EPI (echo planar imaging) images were collected using a 3 T Siemens MAGNETOM Skyra (Erlangen, Germany) fMRI system and a 32-channel head coil. Thirty-two interleaved 3 mm slices (matrix = 64 mm × 64 mm) were collected parallel to the anterior commissure–posterior commissure line with an anterior-to-posterior phase encoding direction (repetition time (TR) = 2000 ms; echo time (TE) = 12, 24.48, 36.96 ms; flip angle = 70°). Additionally, a multi-echo T1-weighted magnetization-prepared rapid gradient-echo (MPRAGE) image (TR = 2530 ms; TE = 1.69, 3.55, 5.41, 7.27 ms; flip angle = 7°) was acquired. T1-weighted MPRAGE images consisted of interleaved 1 mm axial slices (matrix = 256 mm × 256 mm), which were later co-registered to the combined EPI images.

#### fMRI data analysis

fMRI data were analyzed using the Analysis of Functional and Neural Images (AFNI) software package [36]. Multi-echo T1-weighted MPRAGE images were first optimally combined using the “*@compute_OC_weights*” program in AFNI and then processed using *FreeSurfer* (v6.0.0.) [37] to obtain segmentation masks corresponding to the brain (skull-stripped), white matter, and ventricles. The “*@SSwarper*” AFNI program was used for skull-stripping and non-linear warping the combined T1-weighted MPRAGE dataset to standard space using the ICBM 2009c Nonlinear Symmetric atlas [38–40]. The pre-processing was performed on the EPI data using the “*afni_proc.py”* script (see Supplemental Material for additional details). In the first-level analysis, regressors of no interest were created from six motion parameters, per run, fourth-degree polynomial terms to model slow drifts, and shock onsets. Each condition comprising 1-Back/Safe, 1-Back/Threat, 3-Back/Safe, and 3-Back/Threat was entered as a regressor of interest. To determine the differential effect of high (3-Back) compared to low (1-Back) WM load on blood-oxygen-level dependent (BOLD) responses, a general linear test (GLT) was included at the first-level analysis, comparing the 3-Back WM load to the 1-Back WM load. In the second-level analysis, we first performed a general linear model (GLM) with *Drug* (MPH, PLA), *Load* (1-Back, 3-Back), *Condition* (Safe, Threat), and *Run* (Run-1, Run-2) as fixed factors and *Subjects* as random factor. *Sex* and *Age* (grand-mean centered) were added as control covariates. This preliminary second-level analysis was conducted as a quality control to assess the consistency of the main effect of WM load (3-Back vs. 1-Back) with the literature. All subsequent second-level analyses were conducted on the WM load contrast (3-Back vs. 1-Back) included as a GLT in the first-level analysis. Next, we performed a GLM with *Drug* (MPH, PLA), *Condition* (Safe, Threat), and *Run* (Run-1, Run-2) as fixed factors and *Subjects* as random factor to determine their effect on the BOLD responses contrasting the 3-Back with the 1-Back tasks. *Sex* and *Age* (grand-mean centered) were added as control covariates. To better understand this three-way interaction, we further examined the *Drug* × *Condition* interaction in Run-1 and Run-2 separately. The resulting statistical group maps were corrected for multiple comparisons using a cluster-thresholding technique. A minimum cluster size threshold was estimated based on the estimated smoothness of an average of all subjects’ spatial smoothness computed with “*afni_proc.py”* program. Given the non-Gaussian spatial distribution of noise BOLD data, we used an autoregressive function to estimate the smoothness of our fMRI data [41]. A Monte Carlo simulation using the AFNI program “*3dClustSim”* determined the cluster size threshold of 16 voxels (432 μL) corresponding to corrected statistical results with a voxel-wise bi-sided threshold of *p* < 0.001 and with a threshold for whole-brain multiple comparisons set at *p* < 0.05 using NN2 clustering, where clustered voxels can share faces and edges. As complementary analyses, parameter estimates were further extracted from each significant cluster in the *Drug* × *Condition* interaction for Run-1 and Run-2 separately and entered into R. Linear mixed-effects (LME) models were conducted with *Drug* (MPH, PLA), *Run* (Run-1, Run-2), and *Condition* (Safe, Threat) as fixed factors, *Subjects* as random factor, and *Sex* and *Age* (grand-mean centered) included as control covariates.

### *N*-Back WM task performance data analysis

The effects of *Drug* (MPH, PLA), *Condition* (Safe, Threat), *Load* (1-Back, 3-Back), and *Run* (Run-1, Run-2) on cognitive performance (response accuracy and reaction time (RT)) were analyzed with LME using the function “lmer” of the lme4 package in R programming language [42]. *Drug* (MPH, PLA), *Condition* (Safe, Threat), *Load* (1-Back, 3-Back), and *Run* (Run-1, Run-2) were included as fixed-effects factors and *Subjects* as random effect. Additionally, *Sex* and *Age* (grand-mean centered) were added as control covariates (see Supplemental Material for additional details).

### Anxiety subjective rating data analysis

General state anxiety (STAI-s) ratings were analyzed using a LME model with *Drug* (MPH, PLA) and *Time* (T1, T2, and T3) included as fixed-effects factors and *Subjects* as random effect. *Sex* and *Age* (grand-mean centered) were used as control covariates (see Supplemental Material for additional details). Data analysis of anxiety responses during fMRI is presented in Supplemental Material.

## Results

### Sample characteristics

Groups (MPH, PLA) did not differ on age, IQ, weight (kg), shock intensity (mA), or retrospective ratings of shock discomfort following Run-1 and Run-2 (see Table S1).

### WM performance: response accuracy and RT

Results are presented first for accuracy and then for RT (see Tables S2 and S3).

#### Accuracy

The omnibus 4-way (*Drug* × *Run* × *Condition* × *Load*) LME model analysis revealed a trend for the 4-way interaction (*β* = −0.11, *t*_(350)_ = −1.69, *p* = 0.09), in addition to other significant main effects and interactions. Of note, *Drug* exhibited a trend for a main effect, reflecting higher accuracy in the MPH than in the PLA group (*β* = 0.05, *t*_(323)_ = 1.87, *p* = 0.06). Additionally, *Load* significantly affected accuracy with lower accuracy in the 3-Back than in the 1-Back WM task (*β* = −0.17, *t*_(350)_ = −7.57, *p* < 0.001) (see Supplemental Material for results related to the decomposition of the 4-way interaction by run).

#### Reaction time

The omnibus 4-way (*Drug* × *Run* × *Condition* × *Load*) LME model analysis revealed the main effect of *Load* to be statistically significant and the main effect of *Run* to emerge at a trend level. RTs were faster in Run-2 than in Run-1 (*β* = −60.54, *t*_(350)_ = −1.79, *p* = 0.07) and in the 1-Back than in the 3-Back WM task (*β* = −223.41, *t*_(350)_ = −6.59, *p* < 0.001). No other significant effects were found.

### fMRI analysis: whole-brain activation maps

#### Main effect of working-memory load (3-Back vs. 1-Back)—quality-control step

As a quality control to validate the activation pattern of WM, a whole-brain GLM analysis was conducted with *Drug*, *Run*, *Condition*, and *Load* as fixed factors (see Table S4). As expected, the WM-load (3-Back vs. 1-Back) contrast engaged task-positive networks and disengaged the task-negative network (see Fig. S1 and additional details in Supplemental Material). All subsequent analyses were conducted on the WM load contrast (3-Back vs. 1-Back).

#### GLM in whole-brain analysis: *Drug* × *Run* × *Condition* interaction

##### *Drug* × *Run* × *Condition* interaction

At the threshold of *p*_uncorrected_ < 0.01, three clusters reached significance (for a minimal number of voxels equal to *k* = 20). These clusters included regions in the superior parietal lobule (SPL) and the posterior cingulate cortex (PCC)/precuneus (PCU), as well as in the inferior temporal gyrus.

##### *Drug* × *Condition* interaction in Run-1 and Run-2 separately

*Run-1*: *Drug* × *Condition* interaction: At the whole-brain level, five clusters were significant in Run-1 after cluster-wise correction for multiple comparisons (see Table 1). These clusters were located in the parietal/cingulate cortex (left PCU, right PCC, left SPL), prefrontal cortex (right lateral orbitofrontal cortex (lOFC)), and occipital cortex (left lateral occipital cortex (lOC)). This two-way interaction map was decomposed by *Condition*. During Threat, all five clusters were more activated in the MPH group than in the PLA group. During Safe, no clusters significantly differed between the MPH and PLA groups.

**Table 1:** Significant interaction clusters between *Drug* (MPH, PLA) and *Condition* (Safe, Threat) on WM load-related BOLD (3-Back vs. 1-Back) activation in Run-1.

Label	Brodmann area	Side	Peak activation			Number of voxels	Volume (μL)	Mean	SEM	*t* value
			*x*	*y*	*z*					
Drug × Condition in Run-1: MPH vs. PLA in Threat condition										
Precuneus	31	L	−11	−50	38	23	621	4367	184	3.65
Posterior cingulate cortex	31	R	5	−23	44	22	594	3831	162	4.33
Superior parietal	7	L	−17	−80	56	21	567	7910	709	3.51
Lateral occipital	18	L	−20	−104	−5	20	540	3652	253	3.54
Lateral orbitofrontal	47	R	35	38	−17	16	432	4452	275	4.31

Degrees of freedom equal 46. Whole-brain activations are corrected for multiple comparisons using a cluster-based approach with a voxel-wise bi-sided *p* value threshold of *p* < 0.001 and a minimum cluster size of *k* = 16 (432 μL), which corresponds to a cluster-level alpha of *p* < 0.05 using NN2 clustering, where clustered voxels can share faces and edges. LPI means that *x* increases from Left to Right, *y* increases from Posterior to Anterior, *z* increases from Inferior to Superior; Mean and standard error of the mean (SEM) based on absolute value of voxel intensities; correspondences with Brodmann areas are retrieved from the Yale University BioImage Suite.

*L* left, *R* right.

*Run-2*: *Drug* × *Condition* interaction: In Run-2, the *Drug* × *Condition* interaction did not reveal any statistically significant clusters.

#### *Drug* × *Run* × *Condition* interaction on extracted parameter estimates (3-Back vs. 1-Back contrast)

As a reminder, all extracted parameter estimates correspond to the WM load contrast (3-Back vs. 1-Back). Results are illustrated in Fig. 3. As expected, the 3-way *Drug* × *Run* × *Condition* interaction was significant for all clusters. Four of the five clusters exhibited the same pattern of effects. These four clusters included the PCU, PCC, lOC, and lOFC. Figure 3A presents this common pattern using the PCU as the exemplar (see Fig. S2 for the illustration pattern of lOC and lOFC, and Fig. S3 for PCC). The fifth distinct cluster is the SPL whose pattern is illustrated in Fig. 3B.

##### Post hoc analyses of the PCU cluster

The pattern discussed below for the PCU was similar for the other three clusters.

*Run-1*: In Run-1, the PLA group exhibited a significant deactivation to *Load* during Threat (*β* = −0.26, *t*_(365)_ = −4.40, *p* < 0.001) and a non-significant activation to *Load* during Safe (*β* = 0.06, *t*_(365)_ = 1.10, *p* = 0.55). In contrast, the MPH group showed no significant WM-related changes of the PCU responses in either Threat (*β* = 0.09, *t*_(365)_ = 1.55, *p* = 0.49) or Safe (*β* = −0.01, *t*_(365)_ = −0.12, *p* = 0.90). In other words, in Run-1, the PLA group responded to Threat with a significant deactivation of this cluster compared to the MPH group.

*Run-2*: In Run-2, the PLA group exhibited a significant deactivation of the PCU cluster to *Load* during Safe (*β* = −0.16, *t*_(365)_ = −2.72, *p* = 0.03) and an even steeper deactivation during Threat (*β* = −0.26, *t*_(365)_ = −4.32, *p* < 0.001). The MPH group showed a similar, but weaker, pattern. Particularly, the deactivation of the PCU during Safe was not significant (*β* = −0.08, *t*_(365)_ = −1.43, *p* = 0.49), while it was significant during Threat (*β* = −0.17, *t*_(365)_ = −2.89, *p* = 0.02). In other words, in Run-2, *Load* tended to deactivate the PCU in both the PLA and MPH groups and in both Safe and Threat.

Overall, in contrast to Run-1, in Run-2 *Threat* modulated the PCU response to *Load* in both the MPH and PLA groups in a similar way (see Fig. 3A).

##### Post hoc analyses of the SPL cluster

*Run-1*: in Run-1, the PLA group exhibited a significant activation to *Load* during Safe (*β* = 0.33, *t*_(365)_ = 3.39, *p* < 0.001) but no significant effect during Threat (*β* = −0.09, *t*_(365)_ = −0.97, *p* = 0.33). In contrast, the MPH group showed increased activation to *Load* during Safe (*β* = 0.22, *t*_(365)_ = 2.28, *p* = 0.07) and even stronger during Threat (*β* = 0.52, *t*_(365)_ = 5.38, *p* < 0.001). In other words, in Run-1, the activation effect of *Load* during Safe was no longer present during Threat in the PLA group. In contrast, the activation effect of *Load* seen during Safe was potentiated during Threat in the MPH group as well as significantly higher than in the PLA group (see Fig. 3B).

*Run-2*: In Run-2, the PLA group exhibited no significant SPL activation to *Load* during *Safe* (*β* = 0.14, *t*_(365)_ = 1.43, *p* = 0.31), while the activation of the SPL cluster to *Load* was significantly increased during Threat (*β* = 0.26, *t*_(365)_ = 2.66, *p* = 0.03). In contrast, the MPH group showed a significant increase of the SPL activation to *Load* during Safe (*β* = 0.37, *t*_(365)_ = 3.82, *p* < 0.001), which was even stronger during Threat (*β* = 0.43, *t*_(365)_ = 4.47, *p* < 0.001). In other words, in Run-2, the SPL in response to *Load* tended to be activated with PLA, particularly during Threat, while with MPH, the SPL significantly responded to *Load* during both Safe and Threat, with stronger effect during Threat.

### Anxiety subjective ratings: general state anxiety (STAI-s)

The LME model with *Drug* and *Time* (T1, T2, T3) as fixed factors showed a significant main effect for *Time* reflecting a significant increase in anxiety from T1 to T3 (*β* = 4.21, *t*_(98)_ = 4.26, *p* < 0.001), a trend for *Drug* × *Time* [T2 vs. T1] interaction (*β* = 2.62, *t*_(98)_ = 1.90, *p* = 0.06), and a significant *Drug* × *Time* [T3 vs. T1] interaction (*β* = 3.40, *t*_(98)_ = 2.45, *p* = 0.02). The main effect of *Time* consisted of a progressive increase in STAI-s scores from T1 to T3. Participants were at the highest level of anxiety at the end of the study visit. The *Drug* × *Time* interaction reflected a steeper increase in STAI-s scores in the MPH group than in the PLA group (see Table S5 and Fig. S4). However, the mean STAI-s scores over the three time points did not differ between the MPH and PLA groups (*β* = 1.76, *t*_(74)_ = 0.95, *p* = 0.35). Results related to anxiety responses during fMRI are presented in Supplemental Material (see Tables A and B).

## Discussion

Interactions between anxiety and cognitive function are well documented, both at the neural and behavioral level. Neuroimaging studies consistently report a distinct engagement of the networks supporting cognitive function and self-referential processes in anxiety [[18, 19], for reviews, see [20, 21]]. However, the pattern of these anxiety-related effects varies, depending on the types of data (e.g., resting state, emotional task-based, cognitive task-based fMRI). Behavioral studies strongly support a central role of WM in anxiety processes [43, 44]. Although the most commonly reported association describes a disruptive effect of anxiety on cognition [e.g., [12, 13]], a promising interaction for treatment rests on the inhibiting effect of cognitive engagement on anxiety [e.g., [5–10]]. Little is known of the neural mechanisms underlying this phenomenon. The present study aimed at examining the neural correlates underpinning the interaction between anxiety and cognition, extending previous behavioral findings to brain function [16]. For this purpose, cognitive function was manipulated pharmacologically, while anxiety was induced by threat of shock during a WM task.

Three alternate hypotheses were tested regarding the neural systems engaged in the effect of MPH on anxiety-by-cognition interactions: (A) *Increased functional dissociation between the FPCN and the DMN* [for a review, see [20]] as reflected by stronger activation of FPCN regions (task-positive network), which favors cognitive performance, and steeper deactivation of DMN regions (task-negative networks), which prevents interference from irrelevant stimuli. (B) *Redistribution of cognitive resources* as indicated by resource allocation switch from the FPCN to the DMN. This resource reallocation underlies MPH-related increased cognitive efficiency (i.e., less FPCN activation required) [22, 23], and DMN increased engagement to process and modulate anxiety (i.e., DMN activation). (C) *Expansion of overall resources* [16] as reflected by the reinforcement of both FPCN and DMN recruitment. Stronger FPCN activation enhances cognitive performance while inhibiting interference from non-relevant stimuli. Activation of the DMN serves to process anxiety [16]. Our findings align with the third prediction, expansion of overall resources. In line with previous reports [e.g., [24, 25]], we also expected the MPH-related effects to be maximal in the most challenging task condition, i.e., during induced-anxiety (Threat condition), novelty (Run-1), and high cognitive difficulty (3-Back). Findings were consistent with this prediction.

Before discussing the neural findings, it is important to verify the successful implementation of the four manipulations (i.e., *Condition*, *Load*, *Run*, and *Drug*). First, performance (RT and accuracy) was significantly reduced in the 3-Back compared to the 1-Back WM task, and brain activation pattern in response to WM was consistent with literature reports [for reviews, see [45–47]]. Second, RT was slower (task more difficult) in Run-1, when the task was novel, than in Run-2. Third, MPH improved cognitive performance (accuracy) across all task conditions compared to PLA.

We first address the neural effects of MPH (vs. PLA) in Run-1, the critical period for the effect of MPH. As a reminder, all fMRI analyses were conducted on the WM load contrast (3-Back vs. 1-Back) to reflect WM-related BOLD activation. This contrast specifically captured the WM process by minimizing the basic sensory-motor information processing that is common to both the 1-Back and 3-Back tasks.

In Run-1, five clusters behaved differently during *threat* and *safety* in function of *Drug* (*Drug* × *Condition* interaction in MPH vs. PLA). These clusters mapped to the posterior node of the FPCN (SPL), the DMN (PCU, PCC, lOFC), and the primary visual cortex (lOC). For clarity, we use the term FPCN in reference to the role of the SPL cluster and DMN in reference to the role of the PCU, PCC, and lOFC clusters, all three clusters exhibiting the same response pattern.

Overall, during the Run-1/Safe condition, findings did not differ between the MPH and PLA groups. It is only during the threat condition (Run-1/Threat) that MPH impacted neural responses to WM differently than PLA. During *safety*, FPCN was significantly activated, while DMN was not significantly affected. During *threat*, FPCN was significantly activated in the MPH group but showed no significant response in the PLA group. Concomitantly, DMN was significantly activated in the MPH group but was deactivated in the PLA group. These findings suggest that, during *threat*, MPH facilitated the recruitment of the FPCN and also maintained the engagement of the DMN, allowing both systems to function at a higher level compared to PLA. This pattern can be interpreted as an overall MPH-related increase of processing resources, allowing both cognitive task and anxiety to be optimally processed. In other words, while performing the task, individuals continue to be aware of potential threat and keep the capacity to appropriately respond to danger.

The behavioral and self-reported data tend to support this interpretation. Accordingly, general state anxiety self-reported in real-time throughout the study visit using the STAI-s instrument showed a steeper increase in anxiety (from Time-1 to Time-3) in the MPH group compared to the PLA group. This result is consistent with a facilitation of anxiety processing that might be conceived as internally directed cognition (e.g., worrisome thoughts) or self-referential processing elicited by the anxiety-provoking environment [for reviews, see [48–50]]. Performance accuracy was overall better in the MPH than in the PLA group, suggesting that the disproportionate increase in anxiety in the MPH compared to that in the PLA group did not affect the processing of WM. Finally, the formulation of the hypothesis of the expandability of processing resources was born out of our previous behavioral study [16]. In line with the present study, our previous study showed an MPH-related 3-Back performance improvement under *threat*, accompanied by an increase in anxiety-potentiated startle, an objective measure of anxiety.

We now address findings of Run-2. In Run-2, the task and task conditions had become familiar to the participants. In this context, MPH and PLA did not differ on either neural or behavioral measures. The general pattern reflects activation of the FPCN and deactivation or no change of the DMN activity. However, it is interesting to note that, upon inspection of Fig. 3, SPL activation tends to be stronger in MPH than in PLA, during both *safety* and *threat*. Conversely, DMN deactivation tends to be weaker in MPH than in PLA, again during both *safety* and *threat*. Although not statistically different, these patterns would align with the notion of MPH facilitating FPCN engagement and releasing the inhibition of the DMN. To test this possibility, the study power would need to be strengthened by raising the sample size, the number of trials, and the MPH dose. Indeed, we selected the MPH PO of 20 mg dose based on experimental studies successfully using this dose [e.g., [23, 51, 52]] and on clinical experience with adult patients treated for attention-deficit hyperactivity disorder [e.g., [30, 53].

The notion of resources expansibility is important because it is in contrast to the widely accepted concept of limited resources. Accordingly, the idea of limited resources is at the core of the most widely held theoretical accounts of the mechanisms underlying the interaction of anxiety with cognitive activity [12]. Our findings challenge this proposition and, in turn, may bring into light some alternate mechanisms that can be exploited for optimizing or developing new therapeutic strategies. The possibility of expansibility of cognitive resources leads to the idea of cultivating the allocation of additional resources for the regulation of excessive anxiety.

This study is not without limitations. First, we did not observe the same pattern of behavioral performance reported in our previous psychopharmacological study. While our previous behavioral findings showed that enhanced cognitive function using MPH had a protective effect against anxiety at the highest load only, our present findings suggest that MPH might enhance cognitive function more globally, that is irrespective of induced-anxiety or WM load. Several factors might account for the differences between studies. Behavioral findings often do not replicate in the MRI environment compared to the clinic. Many contextual aspects differ between the two settings, including performing the task in a supine position, reduced peripheral vision, stress induced by the MRI environment, or MRI noise [for a review, see [54], see also [55, 56]]. In addition, the physiological responses to induced-anxiety using anxiety-potentiated startle reflex could not be collected in the current work. However, the present neural findings support the theory suggested by our previous clinical study according to which boosting cognition using MPH might expand processing resources. The present findings need to be replicated and additional probes of anxiety should be obtained to better characterize the effects of MPH on behavioral aspects of anxiety (e.g., reframing, avoidance). Such data would provide important information to refine interpretation of the neural findings. Second, self-reported ratings of anxiety during the *N*-Back task did not show any drug effects (see Supplemental Material), unlike the MPH-driven potentiation of anxiety assessed by the startle reflex in our previous psychopharmacological study. Even after accounting for the differences in experimental contexts (clinic vs. scanner), the discrepancy of the MPH-driven effects on the physiological (startle reflex in the behavioral study) and on the self-reported anxiety responses (Likert scales in the current study) is not surprising. These two types of responses reflect the function of two separate biological systems, which may be differently sensitive to MPH. Furthermore, the ratings of anxiety may reflect the “stress” induced by both threat of shock and the performance of a difficult task. This combination of two stressful conditions makes it difficult to clearly interpret these self-report measures. Finally, the retrospective evaluation of anxiety is subject to recollection, which introduces measurement errors. Online monitoring of anxiety in the MRI environment, which have their own drawback (e.g., interference with task performance), could be considered in future studies. Third, no venous blood was drawn to quantify plasma concentrations of MPH throughout the study visit. Therefore, we were not able to determine whether plasma drug concentration significantly differed between participants and whether the differential drug effects between Run-1 and Run-2 was dependent on plasma drug concentration. Fourth, while MPH is a reliable experimental tool, treatment of anxiety with stimulants is not indicated. Therefore, it would be important to explore other interventions that could stretch cognitive resources. Fifth, and most importantly, the present findings stand as proof of concept and need to be tested in clinical anxiety. In conclusion, findings of this study support the theory that processing resources are expandable. This is suggested by MPH-related facilitation of the FPCN and DMN recruitment, which underlies, respectively, higher level of cognitive performance and threat processing. The fuller processing of anxiety conceivably permits better regulation of anxiety so as not to interfere with other tasks. Specifically, resources would be sufficient to prevent threat stimuli from interfering with ongoing behavior and, critically, would also permit to respond appropriately to the sudden emergence of danger while being engaged in a complex cognitive activity. The possibility of expanding the limits of processing resources, here pharmacologically, might open new directions for devising interventions to treat anxiety.

## Supplementary information

Supplemental Material

## References

[1] Airaksinen E, Larsson M, Forsell Y (2005). Neuropsychological functions in anxiety disorders in population-based samples: evidence of episodic memory dysfunction. J Psychiatr Res.

[2] Moran TP (2016). Anxiety and working memory capacity: a meta-analysis and narrative review. Psychol Bull.

[3] Robinson O, Vytal K, Cornwell BR, Grillon C (2013). The impact of anxiety upon cognition: perspectives from human threat of shock studies. Front Hum Neurosci.

[4] Eysenck MW, Derakshan N (2011). New perspectives in attentional control theory. Pers Individ Differences.

[5] Balderston NL, Quispe-Escudero D, Hale E, Davis A, O'Connell K, Ernst M (2016). Working memory maintenance is sufficient to reduce state anxiety. Psychophysiology.

[6] Clarke R, Johnstone T (2013). Prefrontal inhibition of threat processing reduces working memory interference. Front Hum Neurosci.

[7] Patel N, Vytal K, Pavletic N, Stoodley C, Pine DS, Grillon C (2016). Interaction of threat and verbal working memory in adolescents. Psychophysiology.

[8] Vytal K, Cornwell B, Arkin N, Grillon C (2012). Describing the interplay between anxiety and cognition: from impaired performance under low cognitive load to reduced anxiety under high load. Psychophysiology.

[9] Vytal KE, Cornwell BR, Letkiewicz AM, Arkin NE, Grillon C (2013). The complex interaction between anxiety and cognition: insight from spatial and verbal working memory. Front Hum Neurosci.

[10] Vytal KE, Arkin NE, Overstreet C, Lieberman L, Grillon C (2016). Induced-anxiety differentially disrupts working memory in generalized anxiety disorder. BMC Psychiatry.

[11] Bishop SJ (2007). Neurocognitive mechanisms of anxiety: an integrative account. Trends Cogn Sci.

[12] Eysenck MW, Calvo MG (1992). Anxiety and performance: the processing efficiency theory. Cogn Emot.

[13] Eysenck MW, Derakshan N, Santos R, Calvo MG (2007). Anxiety and cognitive performance: attentional control theory. Emotion.

[14] Baddeley AD (1992). Working memory. Science.

[15] Baddeley AD (2001). Is working memory still working?. Am Psychol.

[16] Ernst M, Lago T, Davis A, Grillon C (2016). The effects of methylphenidate and propranolol on the interplay between induced-anxiety and working memory. Psychopharmacology.

[17] Fox MD, Raichle ME (2007). Spontaneous fluctuations in brain activity observed with functional magnetic resonance imaging. Nat Rev Neurosci.

[18] Coutinho JF, Fernandesl SV, Soares JM, Maia L, Gonçalves ÓF, Sampaio A (2016). Default mode network dissociation in depressive and anxiety states. Brain Imaging Behav.

[19] Xiong H, Guo R-J, Shi H-W (2020). Altered default mode network and salience network functional connectivity in patients with generalized anxiety disorders: an ICA-based resting-state fMRI study. Evid Based Complement Alternat Med.

[20] Xu J, Van Dam NT, Feng C, Luo Y, Ai H, Gu R (2019). Anxious brain networks: A coordinate-based activation likelihood estimation meta-analysis of resting-state functional connectivity studies in anxiety. Neurosci Biobehav Rev.

[21] Sylvester CM, Corbetta M, Raichle ME, Rodebaugh TL, Schlaggar BL, Sheline YI (2012). Functional network dysfunction in anxiety and anxiety disorders. Trends Neurosci.

[22] Mehta MA, Owen AM, Sahakian BJ, Mavaddat N, Pickard JD, Robbins TW (2000). Methylphenidate enhances working memory by modulating discrete frontal and parietal lobe regions in the human brain. J Neurosci.

[23] Volkow ND, Fowler JS, Wang GJ, Telang F, Logan J, Wong C (2008). Methylphenidate decreased the amount of glucose needed by the brain to perform a cognitive task. PLoS ONE.

[24] Müller U, Suckling J, Zelaya F, Honey G, Faessel H, Williams SC (2005). Plasma level-dependent effects of methylphenidate on task-related functional magnetic resonance imaging signal changes. Psychopharmacology.

[25] Elliott R, Sahakian BJ, Matthews K, Bannerjea A, Rimmer J, Robbins TW (1997). Effects of methylphenidate on spatial working memory and planning in healthy young adults. Psychopharmacology.

[26] First MB (2002). The DSM series and experience with DSM-IV. Psychopathology.

[27] Wechsler D. Manual for the Wechsler abbreviated intelligence scale (WASI). San Antonio, TX: The Psychological Corporation; 1999.

[28] Pauls AM, O'Daly OG, Rubia K, Riedel WJ, Williams SC, Mehta MA (2012). Methylphenidate effects on prefrontal functioning during attentional-capture and response inhibition. Biol Psychiatry.

[29] Moeller SJ, Honorio J, Tomasi D, Parvaz MA, Woicik PA, Volkow ND (2014). Methylphenidate enhances executive function and optimizes prefrontal function in both health and cocaine addiction. Cereb Cortex.

[30] Gualtieri CT, Wargin W, Kanoy R, Patrick K, Shen CD, Youngblood W (1982). Clinical studies of methylphenidate serum levels in children and adults. J Am Acad Child Psychiatry.

[31] Wargin W, Patrick K, Kilts C, Gualtieri CT, Ellington K, Mueller RA (1983). Pharmacokinetics of methylphenidate in man, rat and monkey. J Pharmacol Exp Therapeutics.

[32] Nandam LS, Hester R, Bellgrove MA (2014). Dissociable and common effects of methylphenidate, atomoxetine and citalopram on response inhibition neural networks. Neuropsychologia.

[33] Balderston NL, Vytal KE, O'Connell K, Torrisi S, Letkiewicz A, Ernst M (2017). Anxiety patients show reduced working memory related dlPFC activation during safety and threat. Depress Anxiety.

[34] Braver TS, Cohen JD, Nystrom LE, Jonides J, Smith EE, Noll DC (1997). A parametric study of prefrontal cortex involvement in human working memory. Neuroimage.

[35] Spielberger CD, Gorsuch RL, Lushene RE, Vagg PR, Jacobs GA. Manual for the state-trait anxiety inventory. Palo Alto, CA: Consulting Psychologists Press; 1983.

[36] Cox RW (1996). AFNI: software for analysis and visualization of functional magnetic resonance neuroimages. Computers Biomed Res.

[37] Morey RA, Petty CM, Xu Y, Hayes JP, Wagner HR, Lewis DV (2009). A comparison of automated segmentation and manual tracing for quantifying hippocampal and amygdala volumes. Neuroimage.

[38] Fonov V, Evans A, McKinstry R, Almli C, Collins D (2009). Unbiased nonlinear average age-appropriate brain templates from birth to adulthood. Neuroimage.

[39] Fonov V, Evans AC, Botteron K, Almli CR, McKinstry RC, Collins DL (2011). Unbiased average age-appropriate atlases for pediatric studies. Neuroimage.

[40] Collins DL, Zijdenbos AP, Baaré WFC, Evans AC. ANIMAL+INSECT: improved cortical structure segmentation. In: Kuba A, Šáamal M, Todd-Pokropek A, editors. Biennial international conference on information processing in medical imaging. Berlin: Springer; 1999. p. 210–23.

[41] Cox RW, Chen G, Glen DR, Reynolds RC, Taylor PA (2017). FMRI clustering in AFNI: false-positive rates redux. Brain Connectivity.

[42] Bates D, Mächler M, Bolker B, Walker S. Fitting linear mixed-effects models using lme4. J Statistical Software. 2015;67:1–48. Accessed on October 16, 2021 from: https://www.jstatsoft.org/index.php/jss/article/view/v067i01.

[43] Rapee RM (1993). The utilisation of working memory by worry. Behav Res Ther.

[44] Stout DM, Bomyea J, Risbrough VB, Simmons AN (2020). Aversive distractors modulate affective working memory in frontoparietal regions. Emotion.

[45] Linden DEJ (2007). The working memory networks of the human brain. Neuroscientist.

[46] Owen AM, McMillan KM, Laird AR, Bullmore E (2005). N‐back working memory paradigm: a meta‐analysis of normative functional neuroimaging studies. Hum Brain Mapp.

[47] Nee DE, Brown JW, Askren MK, Berman MG, Demiralp E, Krawitz A (2013). A meta-analysis of executive components of working memory. Cereb Cortex.

[48] Crittenden BM, Mitchell DJ, Duncan J (2015). Recruitment of the default mode network during a demanding act of executive control. eLife.

[49] Smith V, Mitchell DJ, Duncan J (2018). Role of the default mode network in cognitive transitions. Cereb Cortex.

[50] Uddin LQ, Kelly AM, Biswal BB, Castellanos FX, Milham MP (2009). Functional connectivity of default mode network components: correlation, anticorrelation, and causality. Hum Brain Mapp.

[51] Tomasi D, Volkow ND, Wang GJ, Wang R, Telang F, Caparelli EC (2011). Methylphenidate enhances brain activation and deactivation responses to visual attention and working memory tasks in healthy controls. Neuroimage.

[52] Volkow ND, Wang GJ, Fowler JS, Telang F, Maynard L, Logan J (2004). Evidence that methylphenidate enhances the saliency of a mathematical task by increasing dopamine in the human brain. Am J Psychiatry.

[53] Frölich J, Banaschewski T, Döpfner M, Görtz-Dorten A (2014). An evaluation of the pharmacokinetics of methylphenidate for the treatment of attention-deficit/hyperactivity disorder. Expert Opin Drug Metab Toxicol.

[54] Meléndez JC, McCrank E (1993). Anxiety-related reactions associated with magnetic resonance imaging examinations. JAMA.

[55] Tessner KD, Walker EF, Hochman K, Hamann S (2006). Cortisol responses of healthy volunteers undergoing magnetic resonance imaging. Hum Brain Mapp.

[56] van Maanen L, Forstmann BU, Keuken MC, Wagenmakers EJ, Heathcote A (2016). The impact of MRI scanner environment on perceptual decision-making. Behav Res Methods.

