# Supplementary material for: Methylphenidate modulates interactions of anxiety with cognition
Source: Transl Psychiatry. 2021 Oct 21;11:544. doi: 10.1038/s41398-021-01621-2 (PMC8531440; doi:10.1038/s41398-021-01621-2)
Supplement: Supplementary file 1 — Supplemental Material [file 41398_2021_1621_MOESM1_ESM.docx]

**Supplemental Material**

**Methylphenidate modulates interactions of anxiety with cognition**

C. Gaillard, T. R. Lago, A. X. Gorka, N. L. Balderston, B. Fuchs, R. C. Reynolds, C. Grillon, & M. Ernst

**Supplementary Methods and Material**

Study Procedure

After an initial telephone screen, subjects visited the NIH Clinical Center for a comprehensive screening by a trained clinician. During this screening visit, medical history was obtained, and both a physical exam and the Structured Clinical Interview for DSM-IV using the SCID-I/NP were conducted. On the day of study visit, participants were asked to discontinue eating at least 45 minutes prior to their arrival to the NIH clinical center. Upon arrival to the clinical center (60 minutes pre-drug administration), participants were explained the study and signed the informed consent form approved by the National Institute of Mental Health (NIMH) Combined NeuroScience Institutional Review Board (CNS IRB). A first set of baseline vital signs were then collected by a nurse prior to the drug administration. The drug was administered 90 minutes before starting the working memory (WM) task in the scanner and was immediately followed by the completion of a questionnaire assessing participant’s current state of anxiety [STAI-s T1; 1]. Approximately 30 minutes following drug administration, vital signs were collected for the second time and participants practiced the WM task. Participants were then escorted to the scanning suite. Prior to entering the scanning room, participants self-reported a second time their level of state anxiety (STAI-s, T2). Once in the scanning room, participants underwent a shock work-up procedure and were prepped for scanning. The scanning session began with the collection of a T1-weighted structural scan and a resting state echo-planar images (EPI) data [see 2]. Next, two runs of EPI WM task scans were collected with the first run starting ~90 minutes after drug administration. Right before starting the WM task, participants rated their subjective level of anxiety (baseline). Following the completion of the two WM task runs, participants were asked to evaluate their level of anxiety as a function of task conditions (i.e., 1-Back/Safe, 3-Back/Safe, 1-Back/Threat, 3-Back/Threat). After being removed from the scanner, subjects self-reported for the third time their level of state anxiety (STAI-s, T3). Before discharge, a third set of vitals were collected, and a clinician-administered debriefing medical questionnaire was completed.

Stimuli and apparatus

Stimuli were presented to participants using the Presentation software package (version 14.6, Neurobehavioral Systems, Berkeley, CA) via a rear projection system. In the MRI scanner, participant’s responses were collected using a 4-button fiber optic response device (Current Designs, Philadelphia, PA). Unpredictable electrical shocks delivered in the Threat condition of the WM task were administered via a Digitimer constant current stimulator (DS7A; Digitimer, Letchworth Garden City, UK). The 100 ms shocks were delivered to the subject’s left non-dominant wrist using 2 Ag/AgCl MRI-compatible disposable sticker electrodes. The DS7A was set to deliver a shock intensity ranging from 0 to 100 mA. Shock delivery was individually calibrated before starting the experimental task using a standardized shock work-up procedure.

Shock intensity calibration

Prior to the *N*-Back WM task, participants completed a shock work-up procedure to determine the individual shock intensity level that was appropriate for every subject. The shock level intensity was titrated to be experienced as “highly uncomfortable and aversive, but not painful” by the subject, as assessed on a 10-point Likert scale ranging from 1 (“not at all aversive, maybe you barely felt it”) to 10 (“being highly uncomfortable, but tolerable”). Retrospective shock discomfort was assessed following both runs of the *N*-Back WM task by retrospective rating of “how unpleasant were the electric shocks” on a 11-point Likert scale ranging from 0 (not at all) to 10 (extremely).

Anxiety response during fMRI

Anxiety responses during fMRI was assessed retrospectively, although while in the scanner, via 10-point Likert scales. These ratings were collected after each run of the *N*-Back WM task, and covered anxiety responses to Safe/1-Back, Threat/1-Back, Safe/3-Back, and Threat/3-Back conditions on a 10-point Likert scale ranging from 1 (“not at all”) to 10 (“extremely”).

Additional information related to *N*-Back working memory task performance data analysis

Accuracy and reaction time (RT) were measured for the last 15 trials out of the 18 trials of each block across the 1-Back and 3-back conditions, given that performance in the 3-Back condition becomes meaningful only after that the presentation of the first three trials. Furthermore, trials in which participants received an electrical shock, as well as the three following trials, were discarded to compute accuracy and RT. Finally, RT was measured on correct trials only and on trials in which RT were within the range of 3 standard deviation above or below the mean RT. Satterthwaite approximation implemented in the “*lmerTest*” package was used to estimate bi-sided *p*-values. Post-hoc contrasts were further performed to decompose significant interaction effects using the “*emmeans*” package and Holm’s method was applied to adjust *p*-values for multiple comparisons. Assumptions of mixed-effects model including the independence and homogeneity of variance of the residuals, the linearity of the relationship between predictor and response, and assumptions about the distribution of the residuals [3] were checked to ensure the validity of the models tested.

Additional information related to anxiety subjective ratings data analysis

Satterthwaite approximation implemented in the “*lmerTest*” package was used to estimate bi-sided *p*-values. Post-hoc contrasts were further performed to decompose significant interaction effects using the “*emmeans*” package and Holm’s method was applied to adjust *p*-values for multiple comparisons. Assumptions of mixed-effects model including the independence and homogeneity of variance of the residuals, the linearity of the relationship between predictor and response, and assumptions about the distribution of the residuals [3] were checked to ensure the validity of the models tested.

Anxiety responses during fMRI: Data analysis

Akin to the data analysis of *N*-Back WM task performance, the effects of *Drug* (MPH, PLA), *Condition* (Safe, Threat), *Load* (1-Back, 3-Back), and *Run* (Run-1, Run-2) on self-reported anxiety were analyzed with linear mixed-effects (LME) models using the function “lmer” of the lme4 package in R programming language [4]. *Drug* (MPH, PLA), *Condition* (Safe, Threat), *Load* (1-Back, 3-Back), and *Run* (Run-1, Run-2) were included as fixed effects factors, and *Subjects* as random effect. Additionally, *Sex* and *Age* (grand-mean centered) were added as control covariates. Satterthwaite approximation implemented in the “*lmerTest*” package was used to estimate bi-sided *p*-values. Post-hoc contrasts were further performed to decompose significant interaction effects using the “*emmeans*” package and Holm’s method was applied to adjust *p*-values for multiple comparisons. Assumptions of mixed-effects model including the independence and homogeneity of variance of the residuals, the linearity of the relationship between predictor and response, and assumptions about the distribution of the residuals [3] were checked to ensure the validity of the models tested.

Additional information related to fMRI data acquisition and analysis:

Prior to the first functional task-based run, we acquired two additional sets of 10 multi-echo EPI images using the same parameters, with one “forward” series using the same phase-encoding gradient (anterior-to-posterior phase encoding direction) and the second “reverse” series using a reverse phase-encoding gradient with opposite polarity (posterior-to-anterior phase encoding direction). These additional series were first optimally combined using the “*@compute_OC_weights*” program in AFNI and used to correct for EPI spatial distortion related to phase-encoding direction.

Visual inspection of EPI images confirmed good image quality. The pre-processing was performed on the EPI data using the “*afni_proc.py”* script with the following steps: despiking the time-series (*despike*), slice timing correction (*tshift*), co-registration of the subject’s structural dataset to the EPI dataset (*align*), registration of each volume to the minimum outlier volume and registration of the EPI volumes to subject’s structural dataset (*volreg*), creating a 1-voxel dilated brain mask per run (*mask*), combining echoes into one per run using optimally combined method (*combine*), spatially smoothing within a brain mask using a 6-mm FWHM Gaussian filter (*blur*), scaling each voxel to a mean of 100 (*scale*), and performing the General Linear Model (GLM) analysis (*regress*). All registration operations were combined to avoid repeated resampling of the EPI. The single subject linear regress models were adjusted for motion by censoring EPI volumes and their preceding volume where the derivative of the motion regressors had an Euclidean norm above 0.3 mm. Volumes with more than 5% voxel outliers were censored, as well as time points during which a subject received an electrical shock.

**Supplementary Results**

Additional Results: *N*-Back WM task - *Drug* by *Condition* by *Load* interaction effect on accuracy

Because of the foundational theoretical background of this study, the 4-way interaction was decomposed by *Run*, into two 3-way interactions (*Drug* x *Condition* x *Load*) for each run separately.

*Run-1*: The 3-way (*Drug* x *Condition* x *Load*) linear mixed-effects model analysis revealed a significant 3-way interaction (β = -0.09, *t*_(150)_= -2.09, *p* = 0.04), a *Drug* x *Load* 2-way interaction (β = 0.08, *t*_(150)_= 2.59, *p* = 0.01) and a main effect of *Drug* (β = 0.05, *t*_(187)_= 2.02, *p* = 0.045). Figure A illustrates the *Drug* x *Condition* x *Load* 3-way interaction effect. None of the post-hoc pairwise comparisons performed on the 3-way interaction effect were significant, except for the significant effect of *Load* present in each modality of *Drug* and *Condition.* Both the MPH group and PLA group exhibited lower accuracy for 3-Back than 1-Back at safety ([MPH]: β = -0.25, *t*_(156)_= -11.20, *p* < 0.001 ; [PLA]: β = -0.17, *t*_(156)_= -7.61, *p* < 0.001) and under threat ([MPH]: β = -0.20, *t*_(156)_= -8.86, *p* < 0.001 ; [PLA]: β =-0.21 , *t*_(156)_= -9.36, *p* < 0.001).


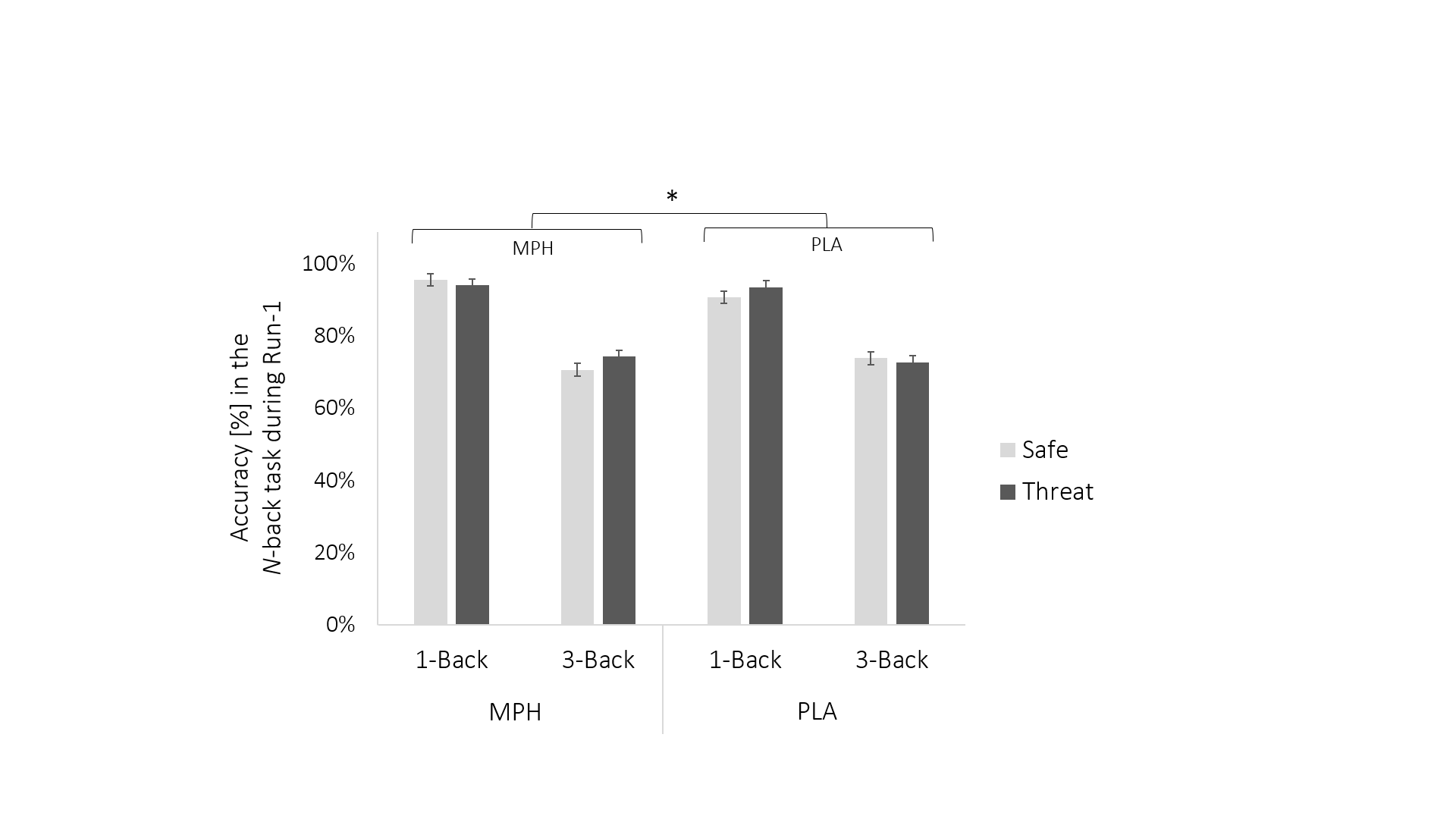


**Fig. A.** Interaction effect of Drug, Condition, and Load on N-Back accuracy performance in Run-1. None of the post-hoc pairwise comparisons performed on the 3-way interaction effect were significant. The main effect of Drug reflected increased accuracy in the methylphenidate (MPH) compared to placebo (PLA) group across all conditions in Run-1. Error bars represent the mean ± standard error of the mean for the linear mixed-effects model comparing Safe condition (light gray bars) to Threat condition (dark gray bars) as a function of Load, i.e. under low WM load (1-Back) and high WM load (3-Back).

*Run-2***:** The 3-way (*Drug* x *Condition* x *Load*) linear mixed-effects model analysis failed to reveal any significant or trends in any of the main effects or interaction effects, except for the main effect of *Load* exhibiting lower accuracy in 3-Back than 1-Back (β = -0.19, *t*_(150)_ = -8.40, *p* < 0.001).

Additional information related to the fMRI analysis: whole-brain activation maps

*Quality-control step - Main effect of working-memory load (3-Back vs. 1-Back):* The task-positive networks included the Fronto-Parietal Control Network (FPCN; left and right rostral middle frontal gyrus, right inferior parietal lobule), the sensory motor network (right thalamus), the basal ganglia (left caudate nucleus), and the visuo-perceptual network (left fusiform gyrus/inferior temporal). The task-negative network consisted of the Default Mode Network (DMN; right lateral OFC, left superior medial OFC, left PCC, and left middle temporal gyrus), the salience network (right anterior insula), and the visual regions (left/right lateral occipital gyri) (See Figure S.1 in Supplemental Material).

Additional Results: Anxiety responses to the task

The omnibus 4-way (*Drug* x *Run* x *Condition* x *Load*) LME model analysis revealed that *Drug* had no effects on anxiety responses to the task conditions, either as a main effect or in interaction with the other factors (see Tables A and B). As illustrated in Figure B and Table B presented below, there was however a main effect of *Condition* (β = 3.64, *t*_(350)_ = 8.85, *p*< 0.001) and of *Load* (β = 2.16, *t*_(350)_ = 5.25, *p*< 0.001).


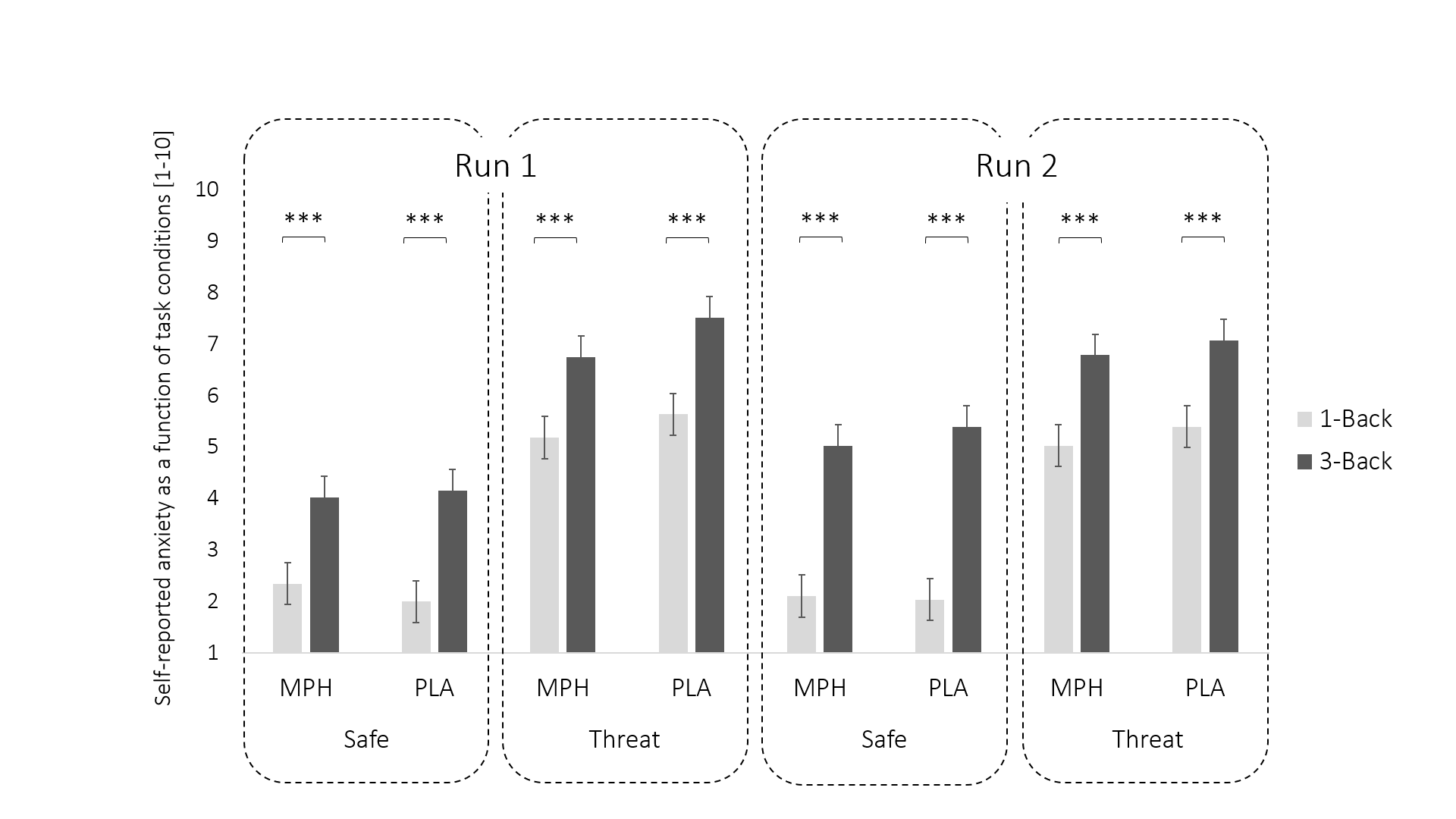


**Fig. B.** Self-reported anxiety during the N-back task as a function of task conditions. Anxiety was analyzed as a function of Drug (MPH, PLA), Run (Run-1, Run-2), Condition (Safe, Threat), and Load (1-Back, 3-Back), with Subjects as random factor, and Sex, Age, and the level of anxiety (Baseline anxiety) experienced prior to the task inserted as covariates. Higher WM-load (3-Back) compared to lower WM-load (1-Back) increased self-reported anxiety across Drug (MPH, PLA), Load (1-Back, 3-Back), and Run (Run-1, Run-2) conditions. Additionally, self-reported anxiety was significantly higher in the Threat compared to Safe conditions across Drug (MPH, PLA), Load (1-Back, 3-Back), and Run (Run-1, Run-2) conditions. The level of anxiety experienced prior to the task was significantly modulating self-reported anxiety during the N-back task, such that higher baseline anxiety was associated to higher self-reported anxiety across all conditions during the N-back task. Error bars represent the mean ± standard error of the mean for the linear mixed-effects model comparing the subjects with placebo (PLA) to subjects with methylphenidate (MPH) under low (1-Back, light gray bars) and high (3-Back, dark gray bars) WM load, separately for the Safe condition in Run-1, Threat condition in Run-1, Safe condition in Run-2, and Threat condition in Run-2. ***p < 0.001.

| **Table A**  Anxiety responses to the task as a function of *Drug* (methylphenidate 20mg [MPH] and placebo [PLA]), *Run* (Run-1, Run-2), *Condition* (Safe, Threat), and *Load* (1-Back, 3-Back). | | | | | | | | | | | | | | | | | |
| --- | --- | --- | --- | --- | --- | --- | --- | --- | --- | --- | --- | --- | --- | --- | --- | --- | --- |
|  | Run-1 of the *N*-Back task | | | | | | | |  | Run-2 of the *N*-Back task | | | | | | | |
|  | Safe | | | | Threat | | | |  | Safe | | | | Threat | | | |
|  | 1-Back | | 3-Back | | 1-Back | | 3-Back | |  | 1-Back | | 3-Back | | 1-Back | | 3-Back | |
|  | *M* | *SE* | *M* | *SE* | *M* | *SE* | *M* | *SE* |  | *M* | *SE* | *M* | *SE* | *M* | *SE* | *M* | *SE* |
|  |  |  |  |  |  |  |  |  |  |  |  |  |  |  |  |  |  |
| MPH | 2.4 | 0.4 | 4.0 | 0.4 | 5.2 | 0.4 | 6.8 | 0.4 |  | 2.1 | 0.4 | 5.0 | 0.4 | 5.0 | 0.4 | 6.8 | 0.4 |
| PLA | 2.0 | 0.4 | 4.2 | 0.4 | 5.6 | 0.4 | 7.5 | 0.4 |  | 2.0 | 0.4 | 5.4 | 0.4 | 5.4 | 0.4 | 7.1 | 0.4 |
| Notes. M, Mean; SE, standard error of the mean. | | | | | | | | | | | | | | | | | |

| **Table B**  Main and interaction effects of the linear mixed-effects model on self-reported anxiety in response to each conditions of the *N*-Back task. | | | | | | |  |
| --- | --- | --- | --- | --- | --- | --- | --- |
|  | Drug | Run | Condition | Load | Baseline anxiety | Sex | Age |
| Anxiety | | | | | | |  |
| Main effects | β = 0.35, *t*(155) = 0.61,  *p* = 0.54 | β = 0.04, *t*(350) = 0.10,  *p* = 0.92 | **β = 3.64, *t*(350) = 8.85,**  ***p* < 0.001** | **β = 2.16, *t*(350) = 5.25,**  ***p* < 0.001** | β = 0.31, *t*(50) = 2.45,  *p* = 0.018 | β = 0.24, *t*(50) = 0.59,  *p* = 0.56 | β = 0.01, *t*(50) = 0.23,  *p* = 0.82 |
| × Drug |  | β = -0.28, *t*(350) = -0.48,  *p* = 0.63 | β = -0.80, *t*(350) = -1.38,  *p* = 0.17 | β = -0.48, *t*(350) = -0.83,  *p* = 0.41 |  |  |  |
| × Run |  |  | β = -0.28, *t*(350) = -0.48,  *p* = 0.63 | β = -0.64, *t*(350) = -1.10,  *p* = 0.27 |  |  |  |
| × Condition |  |  |  | β = -0.28, *t*(350) = -0.48,  *p* = 0.63 |  |  |  |
| × Run × Drug |  |  | β = 0.36, *t*(350) = 0.44,  *p* = 0.66 | β = 0.76, *t*(350) = 0.92,  *p* = 0.36 |  |  |  |
| × Run × Condition |  |  |  | β = 0.44, *t*(350) = 0.54,  *p* = 0.59 |  |  |  |
| × Drug × Condition |  |  |  | β = 0.16, *t*(350) = 0.19,  *p* = 0.85 |  |  |  |
| × Run × Drug × Condition |  |  |  | β = -0.36, *t*(350) = -0.31,  *p* = 0.76 |  |  |  |
|  |  |  |  |  |  |  |  |
| *Note.* *t*, Welch two-sample t-test. | | | | | | |  |

**Supplementary Tables**

| **Table S.1**  Demographics of the two drug groups (methylphenidate 20mg [MPH] and placebo [PLA]) | | | | | | | | | |
| --- | --- | --- | --- | --- | --- | --- | --- | --- | --- |
|  | MPH (*n* = 25) | |  | PLA (*n* = 25) | |  |  |  |  |
|  | *mean* | *SD* |  | *mean* | *SD* |  | *t-value* | *df* | *p-value* |
| Age | 29.28 | 7.15 |  | 27.04 | 6.66 |  | 1.15 | 48 | 0.26 |
| Sex (m/f) | 11/14 | |  | 13/12 | |  |  |  |  |
| WASI scores | 117.56 | 10.16 |  | 117.44 | 11.60 |  | 0.04 | 47 | 0.97 |
| Weight (kg) | 76.77 | 16.92 |  | 73.95 | 14.62 |  | 0.61 | 42 | 0.55 |
| Shock (mA) | 9.63 | 8.84 |  | 12.05 | 14.3 |  | -0.72 | 40 | 0.47 |
| Shock rating following Run-1 | 7.92 | 1.68 |  | 7.88 | 1.67 |  | 0.09 | 48 | 0.93 |
| Shock rating following Run-2 | 8.28 | 1.51 |  | 7.92 | 2.16 |  | 0.68 | 43 | 0.50 |
| *Notes*. The shock level intensity was titrated to be experienced as “highly uncomfortable and aversive, but not painful” by the subject using a 10-point Likert scale ranging from 1 (“not at all aversive, maybe you barely felt it”) to 10 (“being highly uncomfortable, but tolerable”). Retrospective shock discomfort was assessed following both runs of the *N*-Back WM task by retrospective rating of “how unpleasant were the electric shocks” on a 11-point Likert scale ranging from 0 (not at all) to 10 (extremely). df, degree of freedom; f, female; m, male; SD, standard deviation; t-value, Welch two-sample t-test; WASI, Wechsler Abbreviated Scale of Intelligence. | | | | | | | | | |

| **Table S.2**  Main and interaction effects of the linear mixed-effects models on response accuracy and reaction time of the *N*-Back task | | | | | | |
| --- | --- | --- | --- | --- | --- | --- |
|  | Drug | Run | Condition | Load | Sex | Age |
| Response accuracy | | | | | | |
| Main effects | **β = 0.05, *t*(323)  = 1.87,**  ***p* = 0.06** | β = 0.03, *t*(350) = 1.25,  *p* = 0.21 | β = 0.03, *t*(350) = 1.27,  *p* = 0.21 | **β = -0.17, *t*(350 ) = -7.57,**  ***p* < 0.001** | β = 0.00, *t*(50) = -0.01,  *p* = 0.99 | β = 0.00, *t*(50) = -0.47,  *p* = 0.64 |
| × Drug |  | β = -0.03, *t*(350) = -1.01,  *p* = 0.31 | β = -0.04, *t*(350) = -1.38,  *p* = 0.17 | **β = -0.08, *t*(350) = -2.53,**  ***p* = 0.01** |  |  |
| × Run |  |  | β = -0.04, *t*(350) = -1.26,  *p* = 0.21 | β = -0.05, *t*(350) = -1.56,  *p* = 0.12 |  |  |
| × Condition |  |  |  | β = -0.04, *t*(350) = -1.23,  *p* = 0.22 |  |  |
| × Run × Drug |  |  | β = 0.05, *t*(350) = 1.04,  *p* = 0.30 | **β = 0.11, *t*(350) = 2.36,**  ***p* = 0.02** |  |  |
| × Run × Condition |  |  |  | β = 0.08, *t*(350) = 1.87,  *p* = 0.06 |  |  |
| × Drug × Condition |  |  |  | **β = 0.09, *t*(350) = 2.04,**  ***p* = 0.04** |  |  |
| × Run × Drug × Condition |  |  |  | β = -0.11, *t*(350) = -1.69,  *p* = 0.09 |  |  |
| Reaction time | | | | | | |
| Main effects | β = -89.21, *t*(85)  = -1.38,  *p* = 0.17 | **β = -60.54, *t*(350) = -1.79,**  ***p* = 0.07** | β =-17.29, *t*(350) = -0.51,  *p* = 0.61 | **β = 223.41, *t*(350) = 6.59,**  ***p* < 0.001** | β = 78.08, *t*(50) = 1.39,  *p* = 0.17 | β = 7.06, *t*(50) = 1.71,  *p* = 0.09 |
| × Drug |  | β = 43.36, *t*(350) = 0.91,  *p* = 0.37 | β = 48.44, *t*(350) = 1.01,  *p* = 0.31 | β = 29.91, *t*(350) = 0.62,  *p* = 0.53 |  |  |
| × Run |  |  | β = 19.61, *t*(350) = 0.41,  *p* = 0.68 | β = -49.18, *t*(350) = -1.03,  *p* = 0.31 |  |  |
| × Condition |  |  |  | β = 26.84, *t*(350) = 0.56,  *p* = 0.58 |  |  |
| × Run × Drug |  |  | β = -22.47, *t*(350) = -0.33,  *p* = 0.74 | β = 16.70, *t*(350) = 0.25,  *p* = 0.81 |  |  |
| × Run × Condition |  |  |  | β = -1.45, *t*(350) = -0.02,  *p* = 0.98 |  |  |
| × Drug × Condition |  |  |  | β = -77.68, *t*(350) = -1.15,  *p* = 0.25 |  |  |
| × Run × Drug × Condition |  |  |  | β = 28.82, *t*(350) = 0.30,  *p* = 0.76 |  |  |
| *Note.* *t*, Welch two-sample t-test. | | | | | | |

| **Table S.3**  *N*-Back working memory task performance as a function of drug groups (methylphenidate 20mg [MPH] and placebo [PLA]), *Run* (Run-1, Run-2), *Condition* (Safe, Threat), and *Load* (1-Back, 3-Back) | | | | | | | | | | | | | | | | | |
| --- | --- | --- | --- | --- | --- | --- | --- | --- | --- | --- | --- | --- | --- | --- | --- | --- | --- |
|  | Run-1 of the *N*-Back task | | | | | | | |  | Run-2 of the *N*-Back task | | | | | | | |
|  | Safe | | | | Threat | | | |  | Safe | | | | Threat | | | |
|  | 1-Back | | 3-Back | | 1-Back | | 3-Back | |  | 1-Back | | 3-Back | | 1-Back | | 3-Back | |
|  | *M* | *SE* | *M* | *SE* | *M* | *SE* | *M* | *SE* |  | *M* | *SE* | *M* | *SE* | *M* | *SE* | *M* | *SE* |
|  |  |  |  |  |  |  |  |  |  |  |  |  |  |  |  |  |  |
| Response accuracy | | | | | | | | | | | | | | | | | |
| MPH | 95.5 | 1.8 | 70.6 | 1.8 | 94.0 | 1.8 | 74.3 | 1.8 |  | 95.1 | 1.8 | 75.8 | 1.8 | 94.3 | 1.8 | 77.8 | 1.8 |
| PLA | 90.9 | 1.8 | 73.9 | 1.8 | 93.7 | 1.8 | 72.9 | 1.8 |  | 93.7 | 1.8 | 71.8 | 1.8 | 92.5 | 1.8 | 75.1 | 1.8 |
|  |  |  |  |  |  |  |  |  |  |  |  |  |  |  |  |  |  |
| Reaction time | | | | | | | | | | | | | | | | | |
| MPH | 698 | 47.3 | 952 | 47.3 | 730 | 47.3 | 932 | 47.3 |  | 681 | 47.3 | 902 | 47.3 | 710 | 47.3 | 907 | 47.3 |
| PLA | 788 | 47.2 | 1011 | 47.2 | 770 | 47.2 | 1021 | 47.2 |  | 727 | 47.2 | 901 | 47.2 | 729 | 47.2 | 929 | 47.2 |
| *Notes.* M, Mean; SE, standard error of the mean. | | | | | | | | | | | | | | | | | |

| **Table S.4**  Significant clusters for the main effects of *Drug* (MPH, PLA), *Condition* (Safe, Threat), *Load* (3-Back, 1-Back), and *Run* (Run-1, Run-2) on BOLD activation. | | | | | | | | | | | | | | | | | | | | | | | | | |  |  |
| --- | --- | --- | --- | --- | --- | --- | --- | --- | --- | --- | --- | --- | --- | --- | --- | --- | --- | --- | --- | --- | --- | --- | --- | --- | --- | --- | --- |
|  | | | |  | |  | Peak activation | | | |  | | |  | | |  | | |  | | |  | | |  |  |
| Label | | | | Brodmann area | | Side | x | y | z | | Number of voxels | | | Volume (μL) | | | Mean | | | SEM | | | t-value | | |  |  |
|  |  |  |  | |  | | | | | | | | | | | | | | | | | | | | |  |  |
| *Main effect of Drug: MPH vs. PLA* | | | | | | | | | | | | | | | | | | | | | | | | | |  |  |
| - | | | |  | | - | - | - | - | | - | | | - | | | - | | | - | | | - | | |  |  |
| *Main effect of Load: 3-Back vs. 1-Back task* | | | | | | | | | | | | | | | | | | | | | | | | | |  |  |
| Rostral middle frontal gyrus | | | | 10 | | R | 32 | 65 | 14 | | 4442 | | | 119934 | | | 7242 | | | 58 | | | 4.29 | | |  |  |
| Inferior parietal lobule | | | | 7 | | R | 32 | -74 | 53 | | 2597 | | | 70119 | | | 9148 | | | 93 | | | 5.84 | | |  |  |
| Posterior cingulate cortex | | | | 6 | | L | -2 | -14 | 47 | | 1509 | | | 40743 | | | 3602 | | | 42 | | | -6.91 | | |  |  |
| Medial orbitofrontal cortex | | | | 10 | | L | -2 | 62 | -5 | | 1287 | | | 34749 | | | 6198 | | | 82 | | | -4.86 | | |  |  |
| Precentral gyrus | | | | 6 | | R | 62 | 2 | 5 | | 1212 | | | 32724 | | | 4225 | | | 53 | | | -5.95 | | |  |  |
| Superior temporal/Insula | | | | 38 | | L | -32 | 8 | -23 | | 927 | | | 25029 | | | 4017 | | | 46 | | | -4.73 | | |  |  |
| Rostral middle frontal gyrus | | | | 10 | | L | -38 | 62 | 11 | | 567 | | | 15309 | | | 8294 | | | 180 | | | 5.29 | | |  |  |
| Precuneus | | | | 23 | | L | -8 | -53 | 29 | | 314 | | | 8478 | | | 4932 | | | 106 | | | -6.30 | | |  |  |
| Middle temporal gyrus | | | | 37 | | R | 62 | -47 | -11 | | 199 | | | 5373 | | | 6317 | | | 188 | | | 8.72 | | |  |  |
| Superior parietal lobule | | | | 19 | | R | 20 | -95 | 29 | | 155 | | | 4185 | | | 5080 | | | 159 | | | -3.79 | | |  |  |
| Inferior temporal gyrus | | | | 37 | | L | -53 | -65 | -17 | | 146 | | | 3942 | | | 5609 | | | 173 | | | 4.62 | | |  |  |
| Lateral occipital | | | | 18 | | L | -20 | -98 | 26 | | 128 | | | 3456 | | | 4160 | | | 139 | | | -5.10 | | |  |  |
| Thalamus | | | | 50 | | R | 5 | -14 | 14 | | 101 | | | 2727 | | | 3464 | | | 100 | | | 3.61 | | |  |  |
| CC central | | | |  | | L | -5 | 2 | 23 | | 68 | | | 1836 | | | 3113 | | | 100 | | | -4.14 | | |  |  |
| Middle temporal gyrus | | | | 21 | | L | -62 | -2 | -14 | | 53 | | | 1431 | | | 3870 | | | 145 | | | -4.07 | | |  |  |
| Lateral orbitofrontal cortex | | | | 47 | | R | 38 | 35 | -17 | | 41 | | | 1107 | | | 3464 | | | 113 | | | -5.92 | | |  |  |
| Caudate nucleus | | | | 48 | | L | -14 | 11 | 2 | | 26 | | | 702 | | | 3167 | | | 100 | | | 5.15 | | |  |  |
| Posterior cingulate cortex | | | | 24 | | L | -2 | 5 | 29 | | 26 | | | 702 | | | 3141 | | | 168 | | | 3.66 | | |  |  |
| Middle temporal gyrus | | | | 38 | | L | -50 | 14 | -35 | | 25 | | | 675 | | | 3669 | | | 123 | | | -5.27 | | |  |  |
| Lateral occipital gyrus | | | | 37 | | R | 50 | -68 | 2 | | 25 | | | 675 | | | 2595 | | | 89 | | | -3.54 | | |  |  |
| Thalamus | | | |  | | R | 14 | -32 | 23 | | 24 | | | 648 | | | 2943 | | | 103 | | | -5.18 | | |  |  |
| Caudate nucleus | | | | 48 | | L | -17 | 5 | 17 | | 22 | | | 594 | | | 3412 | | | 135 | | | 4.91 | | |  |  |
| Fusiform gyrus | | | | 19 | | L | -29 | -80 | -11 | | 18 | | | 486 | | | 1751 | | | 88 | | | -4.40 | | |  |  |
| Lateral occipital gyrus | | | | 18 | | R | 41 | -86 | -5 | | 16 | | | 432 | | | 1790 | | | 82 | | | -3.79 | | |  |  |
| Precuneus | | | | 23 | | R | 11 | -56 | 14 | | 16 | | | 432 | | | 2969 | | | 115 | | | -3.90 | | |  |  |
|  | | | |  | |  |  |  |  | |  | | |  | | |  | | |  | | |  | | |  |  |
| *Main effect of Condition: Threat vs. Safe* | | | | | | | | | | | | | | | | | | | | | | | | | |  |  |
| CC posterior | | | | 30 | | R | 2 | -41 | | 5 | | 658 | | | 17766 | | | 6814 | | | 120 | | | 3.72 | | |  |
| Posterior cingulate cortex | | | | 6 | | R | 2 | 5 | | 44 | | 578 | | | 15606 | | | 9536 | | | 176 | | | 4.65 | | |  |
| Superior frontal gyrus | | | | 10 | | R | 2 | 62 | | 26 | | 246 | | | 6642 | | | 7030 | | | 195 | | | 3.53 | | |  |
| Pars opercularis | | | | 47 | | L | -50 | 20 | | -5 | | 219 | | | 5913 | | | 7458 | | | 156 | | | 3.82 | | |  |
| Supramarginal | | | | 40 | | R | 53 | -32 | | 32 | | 190 | | | 5130 | | | 8280 | | | 207 | | | 5.19 | | |  |
| Rostral middle frontal gyrus | | | | 10 | | L | -29 | 62 | | 20 | | 160 | | | 4320 | | | 6713 | | | 165 | | | 3.57 | | |  |
| Lateral occipital gyrus | | | | 18 | | L | -20 | -104 | | 5 | | 144 | | | 3888 | | | 6592 | | | 187 | | | 4.62 | | |  |
| Supramarginal | | | | 39 | | L | -62 | -41 | | 29 | | 128 | | | 3456 | | | 7335 | | | 211 | | | 5.05 | | |  |
| Lateral occipital gyrus | | | | 17 | | R | 11 | -98 | | -2 | | 116 | | | 3132 | | | 6586 | | | 182 | | | 3.72 | | |  |
| Precentral gyrus | | | | 6 | | R | 44 | 2 | | 56 | | 91 | | | 2457 | | | 5398 | | | 169 | | | 3.99 | | |  |
| Cerebellum cortex | | | |  | | R | 2 | -50 | | 2 | | 63 | | | 1701 | | | 7957 | | | 417 | | | 3.64 | | |  |
| Fusiform gyrus | | | | 37 | | L | -38 | -62 | | -20 | | 42 | | | 1134 | | | 9030 | | | 334 | | | 4.39 | | |  |
| Thalamus | | | | 48 | | L | -11 | -2 | | 11 | | 32 | | | 864 | | | 5976 | | | 152 | | | 3.60 | | |  |
| Superior parietal lobule | | | | 7 | | R | 20 | -50 | | 68 | | 29 | | | 783 | | | 5723 | | | 216 | | | 4.98 | | |  |
| Fusiform gyrus | | | | 19 | | R | 29 | -74 | | -17 | | 16 | | | 432 | | | 5643 | | | 567 | | | 4.09 | | |  |
|  | | | |  | |  |  |  |  | |  | | |  | | |  | | |  | | |  | | |  |  |
| *Main effect of Run: Run-1 vs. Run-2* | | | | | | | | | | | | | | | | | | | | | | | | | | | |
| Inferior parietal lobule | | | | 19 | | R | 41 | -86 | | 20 | | | 46 | | | 1242 | | | 5521 | | | 190 | | | -4.46 | | |
| Precuneus | | | | 23 | | R | 11 | -56 | | 14 | | | 20 | | | 540 | | | 6451 | | | 227 | | | -4.05 | | |
|  | | | |  | |  |  |  | |  | | |  | | |  | | |  | | |  | | |  | | |
| *Note.* Degrees-of-freedom equal 45. Whole-brain activations are corrected for multiple comparisons using a cluster-based approach with a voxel-wise bi-sided *p*-value threshold of *p* < 0.001 and a minimum cluster size of k = 16 (432 μL), which corresponds to a cluster-level alpha of *p* < 0.05 using NN2 clustering, where clustered voxels can share faces and edges. L, left; R, right; LPI means that x increases from Left to Right, y increases from Posterior to Anterior, z increases from Inferior to Superior; Mean and standard error of the mean (SEM) based on absolute value of voxel intensities; μL, microliters. Correspondences with Brodmann areas are retrieved from the Yale University BioImage Suite. | | | | | | | | | | | | | | | | | | | | | | | | | |  |  |

| **Table S.5**  Main and interaction effects of the linear mixed-effects model on general anxiety state experienced during the study visit | | | | | |
| --- | --- | --- | --- | --- | --- |
|  | Drug | Time [T2 vs. T1] | Time [T3 vs. T1] | Sex [Female vs. Male] | Age |
| Main effects | β = 1.76, *t*(74) = 0.95,  *p* = 0.35 | β = 0.47, *t*(98) = 0.49,  *p* = 0.63 | β = 4.21, ***t*(98) = 4.26,**  ***p* < 0.001** | β = 2.02, *t*(50) = 1.22,  *p* = 0.23 | β = -0.09, *t*(50) =  -0.70, *p* = 0.49 |
| × Drug [MPH vs. PLA] |  | **β = 2.62, *t*(98) = 1.90,**  ***p* = 0.06** | β = 3.40, ***t*(98) = 2.45,**  ***p* = 0.02** |  |  |
| *Note.* Age has been grand-mean centered. T1, Time-1, T2, Time-2, T3, Time-3. | | | | | |

**Supplementary Figures**


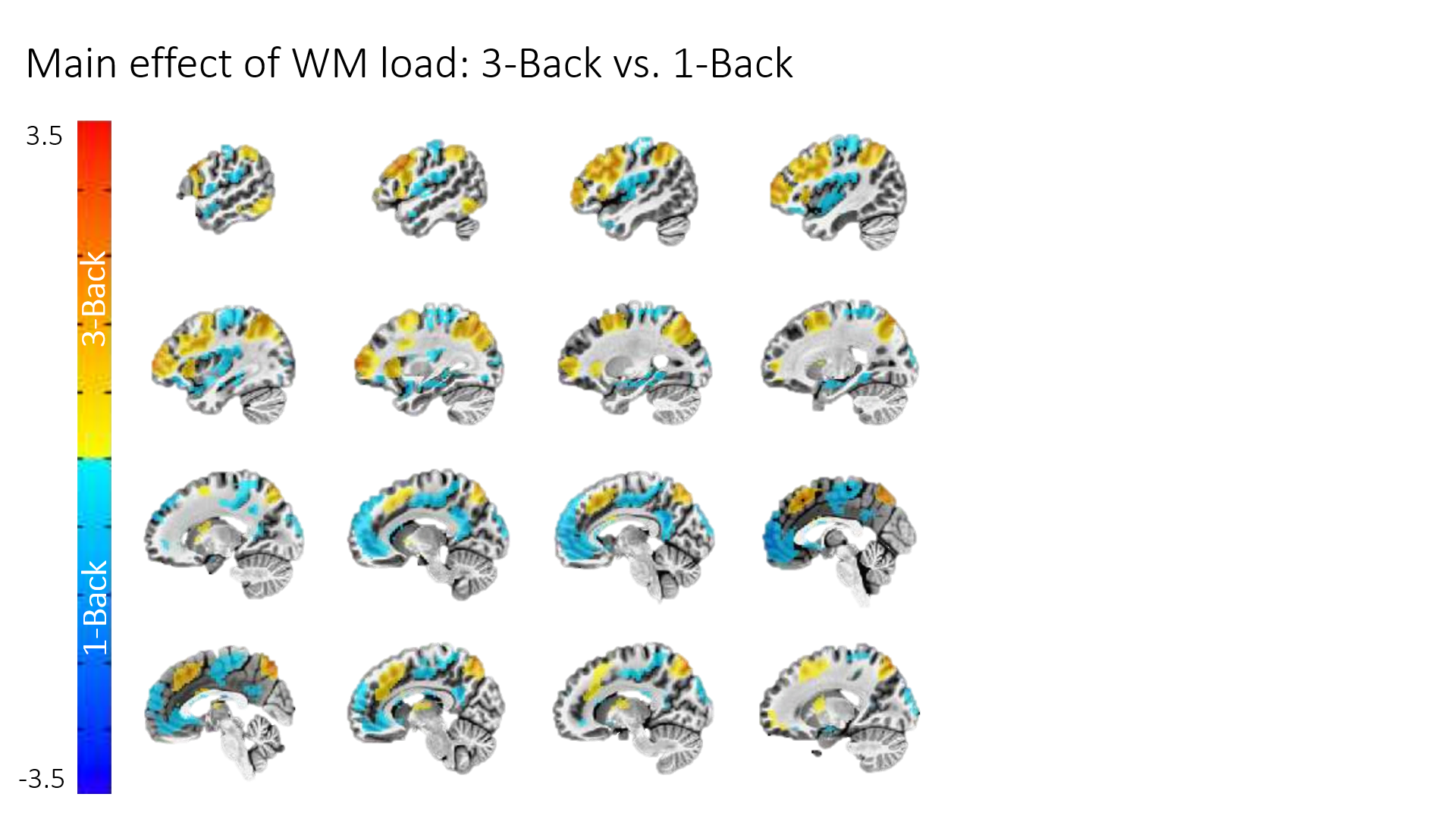


**Fig. S.1.** Main effect of *Load* (3-Back vs. 1-Back) on BOLD activation.

Color bar: Warm colors represent the WM load-related increases (effect size) in BOLD activation, while cool colors represent the WM load-related decreases in BOLD activation. Whole-brain activations are corrected for multiple comparisons using a cluster-based approach with a voxel-wise bi-sided *p*-value threshold of *p* < 0.001 and a minimum cluster size of k = 16 (432 μL), which corresponds to a cluster-level alpha of *p* < 0.05 using NN2 clustering, where clustered voxels can share faces and edges.


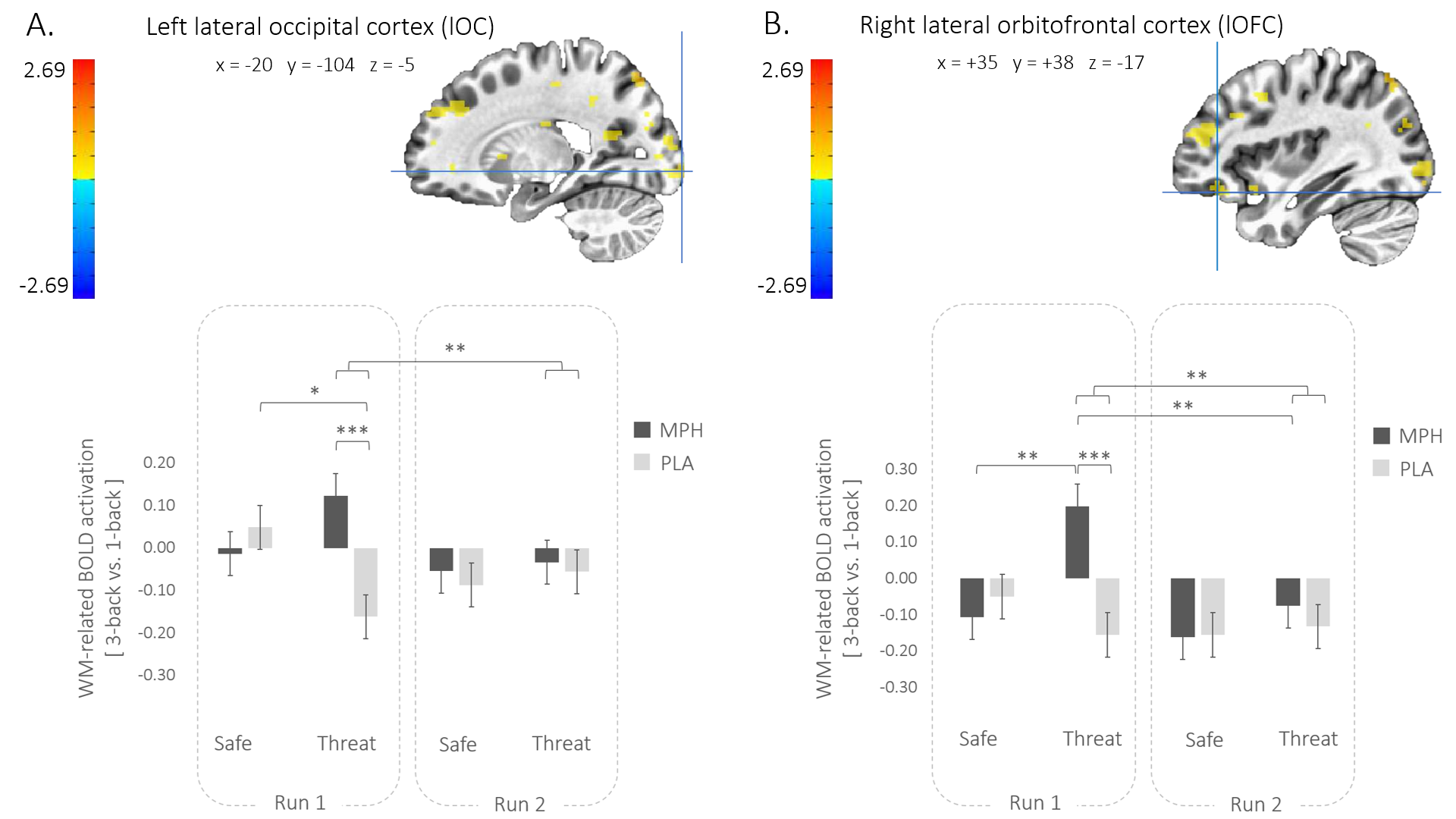


**Fig. S.2**. Interaction effect of *Drug*, *Run* and *Condition* on WM-related BOLD (*3-Back* vs. *1-Back* contrast) on the recruitment of the (A) left lateral occipital (lOC) and (B) right lateral orbitofrontal cortex (lOFC). In the upper panel, the sagittal brain activation maps display the thresholded coefficients activation map of the *Drug* by *Run* by *Condition* interaction in the (A) left lOC and (B) right lOFC. When the task was novel to the participant (Run-1), each cluster exhibited greater WM-related BOLD activation during Threat in the methylphenidate (MPH) group compared to the placebo (PLA) group. In the lower panel, the histograms present the parameter estimates extracted from the (A) left lOC and (B) right lOFC. Linear mixed-effects models were conducted on the parameter estimates with *Drug*, *Run*, and *Condition* as fixed factors, and *Subjects* as random factor. *Sex* and *Age* were included as covariates. Error bars represent the mean ± standard error of the mean.

Color bar: Warm colors represent the WM-related BOLD activation increase (effect size) in MPH compared to PLA, while cool colors represent the WM-related BOLD activation decrease in MPH compared to PLA. Crosshairs depict the location of the voxel with the peak activation for the given cluster. Whole-brain activations are corrected for multiple comparisons using a cluster-based approach with a voxel-wise bi-sided *p*-value threshold of *p* < 0.001 and a minimum cluster size of k = 16 (432 μL), which corresponds to a cluster-level alpha of *p* < 0.05 using NN2 clustering, where clustered voxels can share faces and edges. For visualization purpose, whole-brain activations are thresholded here at *p* = 0.01. **p* < 0.05, ***p* < 0.01, ****p* < 0.001.


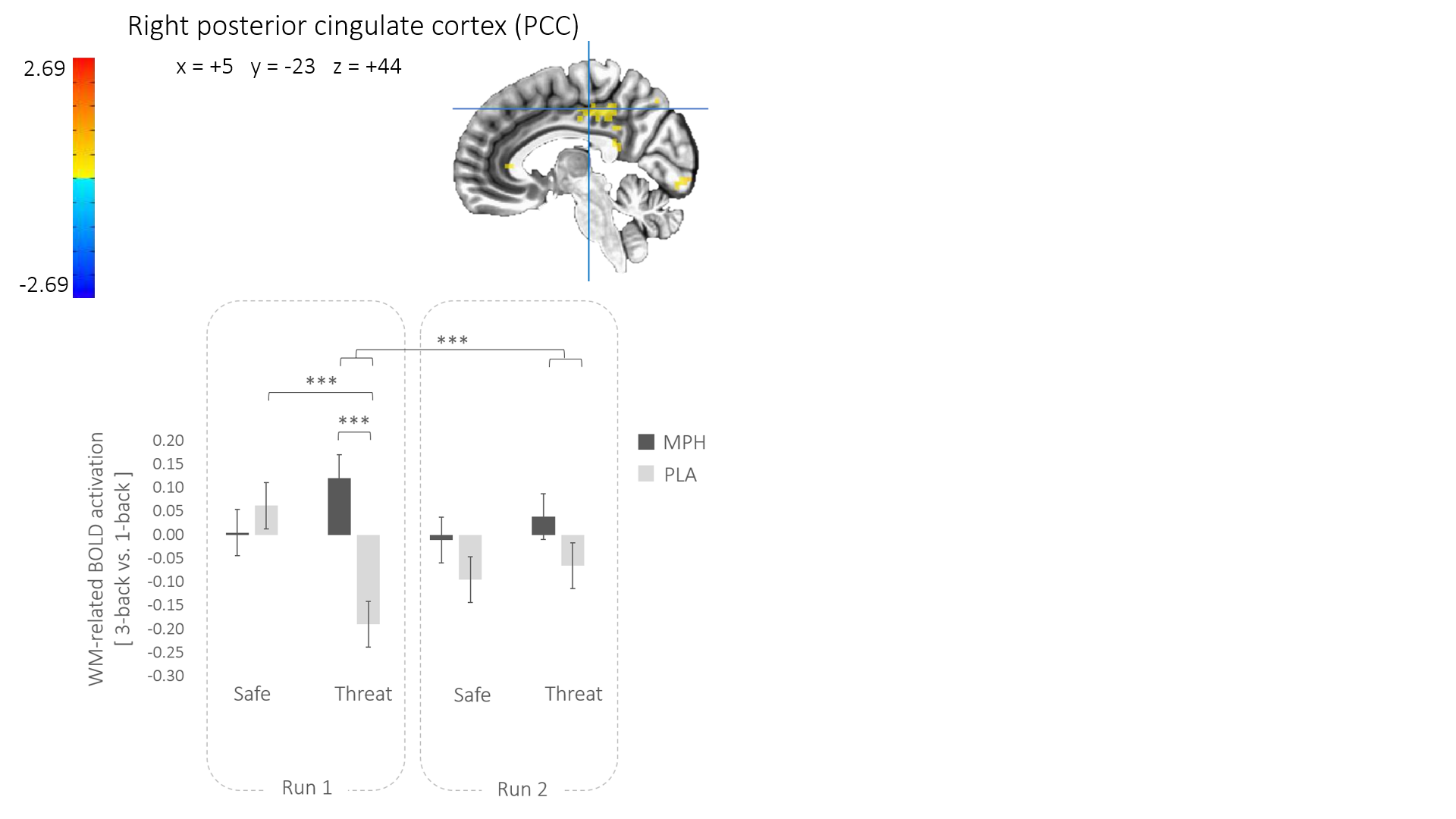


**Fig. S.3.** Interaction effect of *Drug*, *Run*, and *Condition* on WM-related BOLD (3-Back vs. 1-Back contrast) on the recruitment of the right posterior cingulate cortex (PCC) in the Default Mode Network (DMN). In the upper panel, the sagittal brain activation map displays the thresholded coefficients activation map of the *Drug* by *Run* by *Condition* interaction in the PCC. When the task was novel to the participant (Run-1), PCC cluster exhibited greater WM-related BOLD activation during Threat in the methylphenidate (MPH) group compared to the placebo (PLA) group. In the lower panel, the histogram presents the parameter estimates extracted from the PCC. Linear mixed-effects models were conducted on the parameter estimates with *Drug*, *Run*, and *Condition* as fixed factors, and *Subjects* as random factor. *Sex* and *Age* were included as covariates. Error bars represent the mean ± standard error of the mean.

Color bar: Warm colors represent WM-related BOLD activation increase (effect size) in MPH compared to PLA, while cool colors represent WM-related BOLD activation decrease in MPH compared to PLA. Crosshair depicts the location of the voxel with the peak activation. Whole-brain activations are corrected for multiple comparisons using a cluster-based approach with a voxel-wise bi-sided *p*-value threshold of *p* < 0.001 and a minimum cluster size of k = 16 (432 μL), which corresponds to a cluster-level alpha of *p* < 0.05 using NN2 clustering, where clustered voxels can share faces and edges. For visualization purpose, whole-brain activations are thresholded here at *p* = 0.01. **p* < 0.05, ***p* < 0.01, ****p* < 0.001.


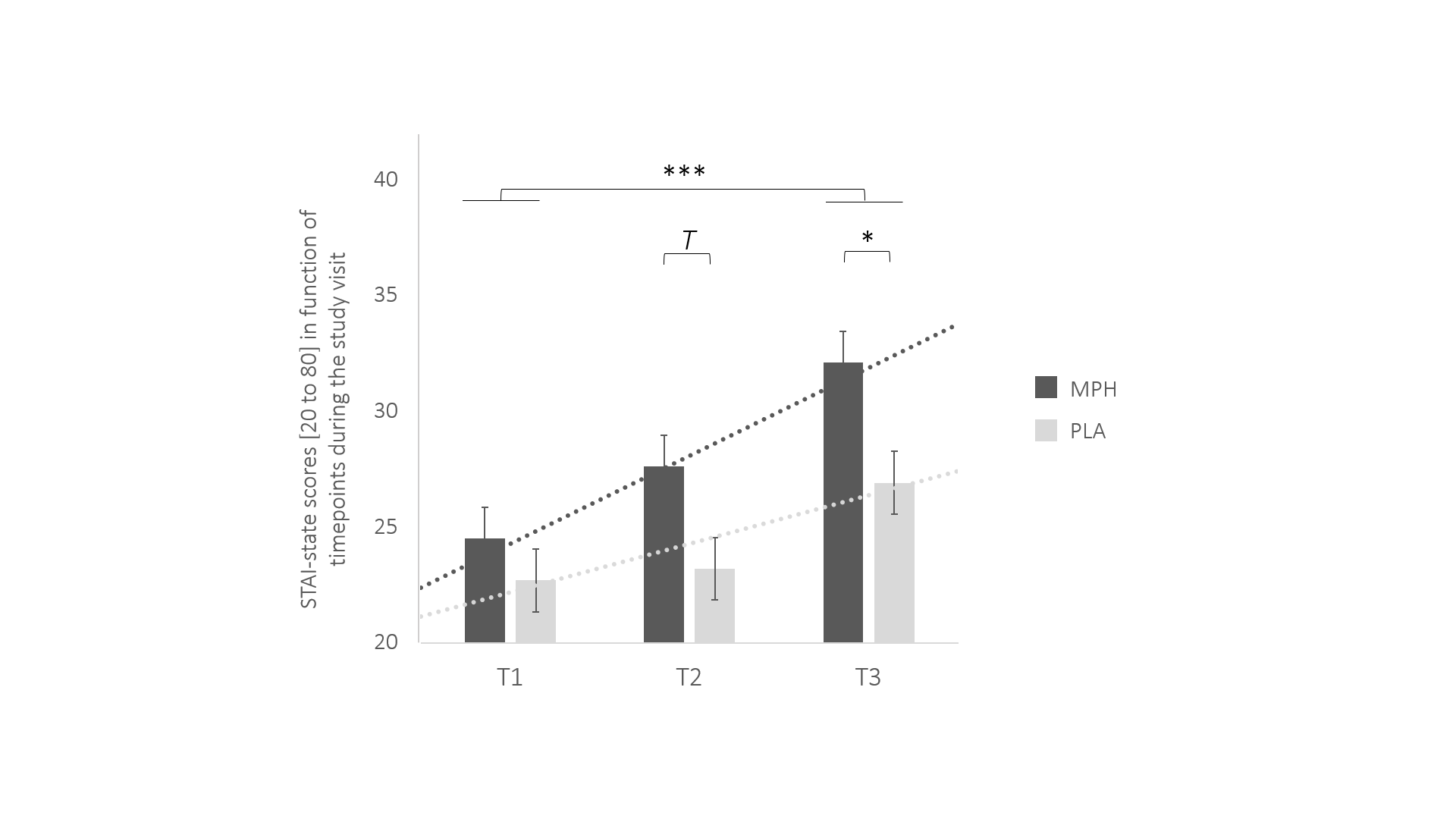


**Fig. S.4.** General anxiety state during the study visit as a function of Time. General anxiety state using the State-Trait Anxiety Inventory [STAI-s; 1] was assessed at three timepoints during the study visit. T1 took place just after drug administration, T2 about 45 minutes following T1 and about 45 minutes prior to scanning session, and T3 after scanning session. Linear mixed-effects models were conducted on general anxiety state with Drug (MPH, PLA) and Time (T1, T2, T3) as fixed factors, Subjects as random factor, and Sex and Age as covariates. General anxiety state significantly increased from T1 to T3 in both methylphenidate (MPH) and placebo (PLA) groups. Compared to participants with PLA, participants with MPH reported a trend for a steeper increase in general state anxiety at T2 compared to T1, as well as a significant steeper increase in general state anxiety at T3 compared T1. Error bars represent the mean ± standard error of the mean for the linear mixed-effects model comparing general anxiety state across time in subjects with PLA (light gray bars) compared to subjects with MPH (dark gray bars). ***p < 0.001, *p < 0.05, ^T^p < 0.10.

**References**

1. Spielberger, C.D., et al., *Manual for the state-trait anxiety inventory Consulting Psychologists Press.* Palo Alto, CA, 1983.

2. Gorka, A.X., et al., *Intrinsic connections between thalamic sub-regions and the lateral prefrontal cortex are differentially impacted by acute methylphenidate.* Psychopharmacology, 2020: p. 1-11.

3. Schielzeth, H., et al., *Robustness of linear mixed-effects models to violations of distributional assumptions.* Methods in Ecology and Evolution, 2020. **11**(9): p. 1141-1152.

4. Bates, D., et al., *lme4: Linear mixed-effects models.* R package version 0.999375-37/r1127. R-Forge, 2010.
